# Validation of Nutritional Approaches to Modulate Cardiovascular and Diabetic Risk Factors in Patients with Hypertriglyceridemia or Prediabetes—The MoKaRi II Randomized Controlled Study

**DOI:** 10.3390/nu16091261

**Published:** 2024-04-24

**Authors:** Theresa S. Braun, Timo Drobner, Kristin Kipp, Michael Kiehntopf, Peter Schlattmann, Stefan Lorkowski, Christine Dawczynski

**Affiliations:** 1 Junior Research Group Nutritional Concepts, Institute of Nutritional Sciences, Friedrich Schiller University Jena, Dornburger Straße 25-29, 07743 Jena, Germany; theresa.braun@uni-jena.de (T.S.B.); timo.drobner@uni-jena.de (T.D.); 2 Competence Cluster for Nutrition and Cardiovascular Health (nutriCARD) Halle-Jena-Leipzig, Dornburger Straße 25-29, 07743 Jena, Germany; peter.schlattmann@med.uni-jena.de (P.S.); stefan.lorkowski@uni-jena.de (S.L.); 3 Department of Pediatrics and Adolescent Medicine, Sophien- and Hufeland Hospital, Henry-van-de-Velde-Str. 1, 99425 Weimar, Germany; k.kipp@klinikum-weimar.de; 4 Institute of Clinical Chemistry and Laboratory Diagnostics, University Hospital Jena, Am Klinikum 1, 07747 Jena, Germany; michael.kiehntopf@med.uni-jena.de; 5 Department of Medical Statistics and Epidemiology, Institute of Medical Statistics, Computer and Data Sciences, University Hospital Jena, Bachstraße 18, 07743 Jena, Germany; 6 Department of Nutritional Biochemistry and Physiology, Institute of Nutritional Sciences, Friedrich Schiller University Jena, Dornburger Straße 25, 07743 Jena, Germany

**Keywords:** cardiovascular risk, hypertriglyceridemia, prediabetes, diabetes mellitus type 2, menu plans, fish oil supplementation

## Abstract

Hypertriglyceridemia and diabetes mellitus type 2 are among the most important metabolic diseases globally. Diet plays a vital role in the development and progression of both clinical pictures. For the 10-week randomized, controlled, intervention study, 67 subjects with elevated plasma triglyceride (TG) concentrations (≥1.7 mmol/L) and 69 subjects with elevated fasting glucose concentrations (≥5.6 < 7.0 mmol/L) were recruited. The intervention groups received specially developed, individualized menu plans and regular counseling sessions to lower (A) TG or (B) fasting glucose and glycated hemoglobin A1c as well as other cardiovascular and diabetic risk factors. The hypertriglyceridemia intervention group was further supplemented with fish oil (3.5 g/d eicosapentaenoic acid + docosahexaenoic acid). The two control groups maintained a typical Western diet. Blood samples were taken every 2 weeks, and anthropometric data were collected. A follow-up examination was conducted after another 10 weeks. In both intervention groups, there were comparable significant reductions in blood lipids, glucose metabolism, and anthropometric parameters. These results were, with a few exceptions, significantly more pronounced in the intervention groups than in the corresponding control groups (comparison of percentage change from baseline). In particular, body weight was reduced by 7.4% (6.4 kg) and 7.5% (5.9 kg), low-density lipoprotein cholesterol concentrations by 19.8% (0.8 mmol/L) and 13.0% (0.5 mmol/L), TG concentrations by 18.2% (0.3 mmol/L) and 13.0% (0.2 mmol/L), and homeostatic model assessment for insulin resistance by 31.8% (1.1) and 26.4% (0.9) (*p* < 0.05) in the hypertriglyceridemia and prediabetes intervention groups, respectively. Some of these changes were maintained until follow-up. In patients with elevated TG or fasting glucose, implementing individualized menu plans in combination with regular counseling sessions over 10 weeks led to a significant improvement in cardiovascular and diabetic risk factors.

## 1. Introduction

Hypertriglyceridemia (fasting triglyceride (TG) serum concentrations: ≥1.7 mmol/L) is one of the most common forms of dyslipidemia and is associated with an increased risk of cardiovascular disease (CVD) [1,2]. Almost one-third of the world’s population has elevated TG concentrations [3,4,5]. More men than women are affected, and the proportion increases with age [3,5,6,7,8]. Due to the increasing prevalence of diseases associated with hypertriglyceridemia, such as overweight and obesity, diabetes mellitus type 2 (DMT2), and metabolic syndrome, the prevalence of hypertriglyceridemia is expected to increase itself [2,9,10,11]. A combination of genetic predisposition (primary factors) and secondary lifestyle-related factors that lead to an increased production or decreased clearance of TG-rich lipoproteins (very low-density lipoprotein (VLDL), chylomicrons) or both can lead to the onset of hypertriglyceridemia [12]. The therapy includes well-defined changes in dietary and lifestyle habits, which could potentially reduce TG by 20–50% [13]. Weight loss, increased physical activity, reduced carbohydrate (CHO) intake, abstinence of alcohol, and an increased intake of *n*-3 long-chain fatty acids eicosapentaenoic acid (EPA) and docosahexaenoic acid (DHA) are recommended [13,14]. Pharmacological therapy is recommended only if lifestyle changes do not lead to a sufficient reduction in TG [14].

DMT2 is one of the most common public health concerns, with a steadily increasing prevalence for many years. According to a recent report by the German Diabetes Society and the German Diabetes Aid, the number of diagnosed cases increased from about 7 million to about 8.7 million between 2015 and 2022 [11]. The statutory health insurance companies estimate that around 9–10% of the adult population is affected [11]. In addition, another 15 to 20 million Germans suffer from prediabetes (glycated hemoglobin A_1c_ (HbA1c) of 5.7–6.4%) [15]. A prevalence of around 537 million people was estimated for 2021 and an increase to around 783 million cases is expected by 2045 [16]. The main risk factors are family predisposition, age, low physical activity, and obesity [17]. The WHO, therefore, prescribes physical activity, a healthy diet, and maintaining or achieving a normal body weight as key measures to prevent and delay the onset of DMT2 [18]. The spectrum of secondary diseases is diverse, with CVD being among the most common consequences and the leading cause of shortened life expectancy in people with DMT2 [19]. In Germany, around 16% of all deaths are attributable to the consequences of DMT2 [20]. Compared to healthy individuals, the mortality risk of patients with DMT2 is 1.5 times higher [21]. Current research activities support nutritional approaches as a therapy focus. However, results are not consistent, as comparable interventions produce contradictory results. Factors such as study duration and the selection of target markers seem to play a central role [22,23,24]. Current nutritional concepts focus, in particular, on improving the quantity and quality of CHO, all with varying degrees of effectiveness [25,26,27].

Since diet modifications are consistently considered to be effective in the prevention and treatment of both hypertriglyceridemia and DMT2 [13,14,28,29], two nutritional concepts have been developed to counteract the mentioned clinical pictures. These two concepts are mainly based on specially developed daily menu plans. The effectiveness of the concepts in comparison to a traditional German Western diet was the focus of the present study.

## 2. Materials and Methods

### 2.1. Study Design

The MoKaRi II study was conducted as a randomized, controlled, single-center intervention study in parallel design. The study was divided into two study arms (hypertriglyceridemia concept and prediabetes concept), each consisting of an intervention and a control group. The study took place in Eastern Germany between April and November 2022. Men and women between 35 and 75 years with a body mass index (BMI) of ≥20 to ≤35 kg/m^2^ and with either elevated TG (≥1.7 mmol/L = hypertriglyceridemia concept; [30]) and/or elevated fasting blood glucose (≥5.6 < 7.0 mmol/L = prediabetes concept; [31]) were enrolled. If a subject had both elevated TG and glucose concentrations at screening, they were assigned randomly to one of the two study arms. In addition, all subjects had to meet the following inclusion and exclusion criteria:

- Consumption of a traditional “Western diet” composed of meat, sausage, dairy products, cereals, vegetables, fruits etc.
- Stable eating habits at least 1 year before enrollment
- No relevant food allergies (e.g., milk, nuts etc.)
- No antihypertensive medication or stable dose for >3 months prior to the start of the study and during the entire study period
- No acute or chronic diseases which could affect the results of the study
- No systemic glucocorticoids or lipid-lowering medication
- No use of dietary supplements, incl. multivitamins, fish oil capsules, minerals, and trace elements 3 months before and during the entire study period
- No weight loss or weight gain (>3 kg) during the last 3 months before study
- No pregnancy or lactation

**Figure 1:**
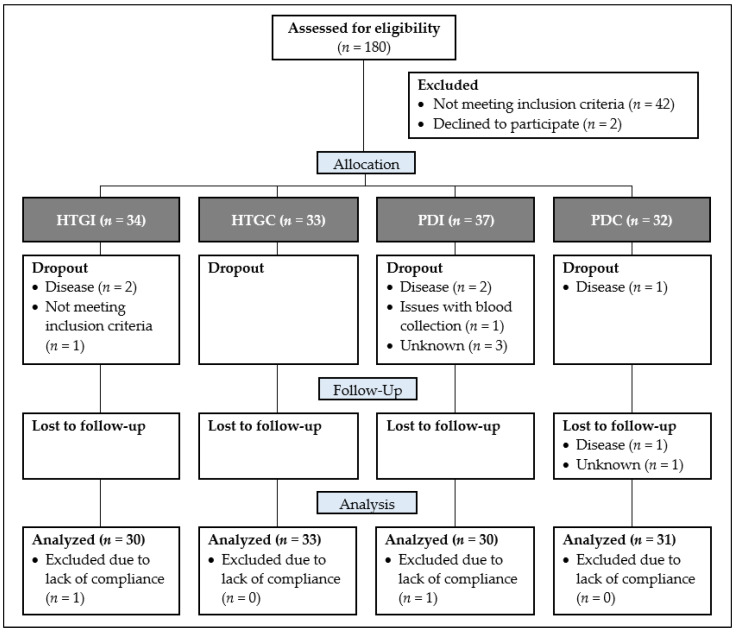
Flowchart diagram of the study population in the different phases of the study. In total, 180 subjects were screened for eligibility for at least one of the two study arms; 44 subjects had to be excluded, so that 67 subjects were randomized to the hypertriglyceridemia study arm and 69 subjects to the prediabetes study arm. After completion of the study, sorted by group, 30, 33, 30, and 31 subjects were included in at least one statistical analysis (subjects lost to the follow-up were included in the analyses of the intervention period). Abbreviations: HTGC, hypertriglyceridemia control; HTGI, hypertriglyceridemia intervention; PDC, prediabetes control; PDI, prediabetes intervention.

Prior to the run-in, 180 subjects were screened for eligibility before enrollment; 44 were excluded as they did not meet the inclusion criteria or declined to participate. After screening, 136 subjects started with the run-in phase and were assigned at random to one of the four study groups based on their eligibility (Figure 1).

**Figure 2:**
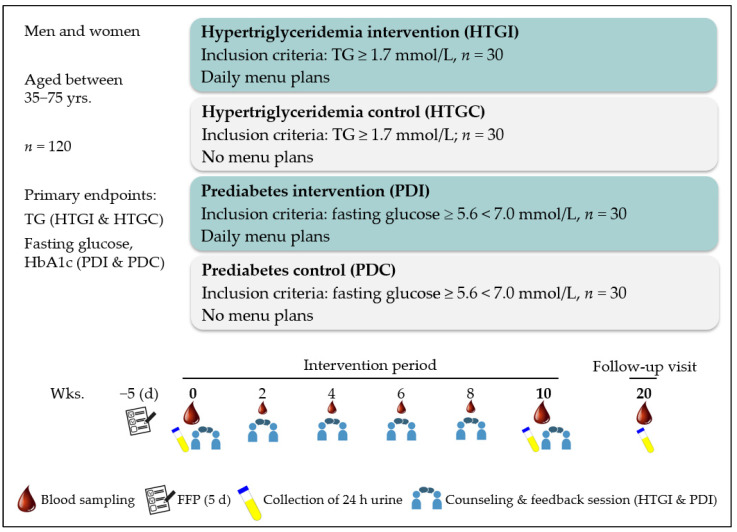
Study design of the MoKaRi II study. Abbreviations: FFP, food-frequency protocol; HbA1c, glycated hemoglobin A_1c_; HTGC, hypertriglyceridemia control; HTGI, hypertriglyceridemia intervention; PDC, prediabetes control; PDI, prediabetes intervention; TG, triglycerides.

The intervention period lasted 10 weeks, with examinations in 2-week intervals. A follow-up visit took place after a further 10 weeks. In addition to regular blood sampling at each visit, the collection of 24-h urine and the completion of various questionnaires were conducted at baseline, after 10 weeks, and at follow-up (Figure 2).

The study’s primary outcome measures were changes in (A) TG (hypertriglyceridemia concept) and (B) fasting glucose, and HbA1c values (prediabetes concept). Secondary outcome measures were anthropometric data, other blood lipids and markers of glucose metabolism, blood pressure, additional cardiovascular risk factors, and nutrient status and intake.

### 2.2. Assessment of Nutritional Habits, Socio-Economic Status and Medication

The participants were required to record their food and beverage intake over a 5-day period before baseline to document variations in dietary patterns within and between the groups. The full self-report of the individual dietary intake was based on the “Freiburger Ernährungsprotokoll Standard” template, which was provided by PRODI version 6.4 (Nutri-Science, Stuttgart, Germany) and included foods, beverages, and typical portion sizes of a common German diet. Foods that were not listed in the template were manually documented by the subjects including the name and the amount of the food consumed. The daily energy and nutrient intake was calculated with the software package PRODI version 6.11. The socioeconomic status of the participants was measured using selected items from the German National Consumption Survey II and the German Health Interview and Examination Survey for Adults. The questionnaire included questions about marital status, household size, educational achievements, income, occupation, and employment status. In addition, subjects filled out questionnaires to assess physical activity as well as health and disease status (including medication use).

### 2.3. Study Diet—The MoKaRi II Concept

**Figure 3:**
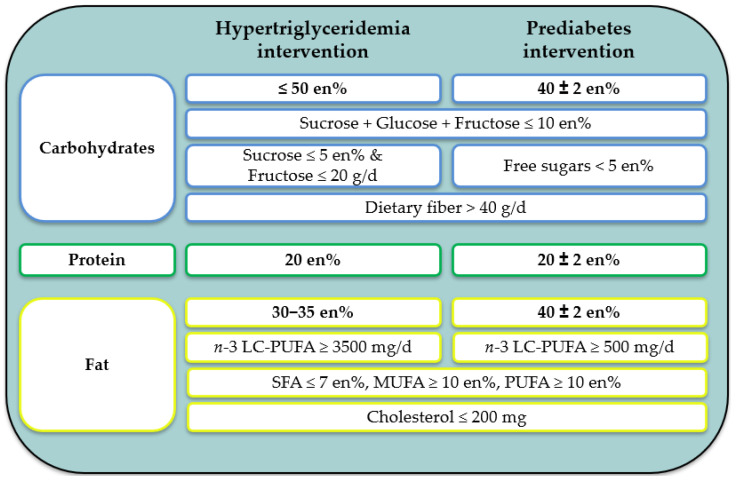
Main criteria of the MoKaRi II menu planes. Abbreviations: en%, percent of daily energy intake; MUFA, monounsaturated fatty acids; LC, long-chain; PUFA, polyunsaturated fatty acids; SFA, saturated fatty acids.

The subjects in both intervention groups implemented their respective nutritional concepts over a 10-weeks intervention period. The concepts were based on daily menu plans that define the entire diet during the intervention period. The menu plans were developed in 11 energy levels between 1700 and 2800 kcal to provide individualized plans according to participant’s energy requirements, which vary depending on age, sex, and physical activity. The macronutrient profile of the menu plans of the hypertriglyceridemia intervention (HTGI) group and the prediabetes intervention (PDI) group is shown in Figure 3.

In addition, the menu plans were characterized by the following criteria:

- Micronutrient intake according to the recommendations of the German Nutrition Society (DGE) (except for vitamin D (which is mainly covered by UVB radiation from sunlight), selenium (as no data on concentrations in food are available in the most recent database used), and iodine (as no iodized salt was specified in the menu plans))
- Reduced intake of salt and absence of alcohol
- Increased consumption of vegetables, fruits, and whole grains
- Reduced consumption of highly processed, calorie-dense, nutrient-poor foods

To increase compliance with the menu plans, subjects were provided with selected commercially available foods such as linseed oil, rapeseed oil, olive oil, and various nuts. Furthermore, subjects in the HTGI group received fish oil capsules (660 mg EPA and 220 mg DHA per gram) to ensure daily intake of ≥3500 mg *n*-3 long-chain polyunsaturated fatty acids (PUFA).

One-to-one interviews were conducted with all subjects of the intervention groups at each visit to the study center. This interview consisted of the following elements:

- Short counselling session on one topic or aspects of a healthy diet (e.g., nuts, berries, legumes, sugar, fats)
- Discussion of the trends of selected study parameters throughout the study (e.g., blood lipids, markers of glucose metabolism)
- Discussion and problem-solving regarding the implementation of the menu plans in everyday life, based on a protocol in which deviations from the menu plans had to be documented. This procedure was also used to verify and ensure the compliance of the subjects.

None of these elements were included in the control groups.

### 2.4. Sample Collection, Parameter Analyses and Further Measurements

Blood glucose was measured during the screening using Contour XT (Bayer, Leverkusen, Germany) and TG using Accutrend Plus (Roche Diagnostics, Mannheim, Germany). At baseline and during the following visits, blood samples were taken by venipuncture between 7:00 a.m. and 11:00 a.m. after an overnight fasting period of at least 12 h. Excessive physical activity and alcohol consumption were not allowed the day before and the morning of the venipuncture. Urine was collected for 24 h before baseline, before the end of the intervention period (week 10), and before the follow-up visit (week 20).

Fasting peripheral venous blood samples were centrifuged to separate erythrocytes, plasma, and serum. The study parameters were either analyzed immediately after blood sampling or urine collection or were stored at −80 °C using aliquots (erythrocytes, serum, plasma) or at −20 °C (24-h urine) until the analysis. All samples were prepared according to standard operation procedures. All study parameters (besides fatty acid distribution in erythrocytes) were analyzed in serum, plasma, and urine at the Institute of Clinical Chemistry and Laboratory Diagnostics, University Hospital Jena, using Cobas 8000 (Roche, Mannheim, Germany), Tosoh HLC-723G11 (Sysmex, Norderstedt, Germany), HPLC (Shimadzu, Kyoto, Japan), or AAS 5 FL (Analytik Jena AG, Jena, Germany) according to the manufacturer’s recommendations (Table S1). The Institute of Nutritional Sciences, Friedrich Schiller University Jena, analyzed fatty acid distribution in erythrocytes. At first, fat was extracted using the Folch and Bligh and Dyer procedures [32,33]. Afterwards, the extracted lipids were saponified and methylated [34]. The success of the methylation was confirmed by separation on silica gel aluminum plates. The resulting fatty acid methyl esters (FAME) were then analyzed via gas chromatography (GC-17V3, Shimadzu, Duisburg, Germany). Quantification of each FAME was calculated using LabSolutions software version 5.92 (Shimadzu, Duisburg, Germany). FAME are presented in relation to the total FAME content.

Anthropometric, blood pressure, and heart rate measurements were always taken by a trained study nurse, with subjects barefoot and in light clothing (single measurement). Waist circumference was measured midway between the lower rib margin and the iliac crest (a thumb’s breadth above the navel). Arterial blood pressure was measured on the upper arm with the subject in sitting position after resting in this posture for at least 10 min. All measurements were conducted using calibrated instruments: Scale with integrated stadiometer (seca813, seca, Hamburg, Germany; only in prediabetes study arm); ergonomic tape measure (seca212, seca, Hamburg, Germany); automatic blood pressure device (boso-medicus uno, BOSCH + SOHN, Jungingen, Germany). Body composition was assessed by using Body Composition Analyzer (seca 515/514, seca, Hamburg, Germany) in the hypertriglyceridemia study arm and by using Body Impedance Analyzer (BIA 2000-S, Data Input, Pöcking, Germany) in the prediabetes study arm.

### 2.5. Statistical Methods

The power analysis is based on the study results of Lee et al. (2016) and Bays et al. (2011) and was performed using the statistical software G*Power version 3.1.9.7 (The G*Power Team, Düsseldorf, Germany) [35,36]. Lee et al. (2016) randomly divided 93 subjects with diagnosed DMT2 into 2 groups (vegan diet, conventional diet recommended by the Korean Diabetes Association). The primary study endpoint was the change in HbA1c values. After 12 weeks, HbA1c values decreased in both groups, with the vegan diet resulting in a higher reduction (−0.9% vs. −0.3%; *p* = 0.010) [35]. Based on these data, a group size of 26 subjects has over 95% power to achieve a difference in HbA1c of 0.6% (SD: 0.9%). Bays et al. (2011) included 229 patients with elevated fasting TG concentrations (≥500 mg/dL and ≤2000 mg/dL) [36]. Patients were randomly divided into three groups (A: 4 g/d of AMR101 (containing ≥ 96% EPA ethyl ester and no DHA or DHA ethyl ester); B: 2 g/d of AMR101; C: placebo). The primary endpoint was the change in TG. After 12 weeks, the baseline TG concentration decreased from 680.0 to 502.0 mg/dL in group A (*p* < 0.001). In the placebo group, the baseline TG concentration increased from 703.0 to 745.5 mg/dL (n.s.) [36]. Based on these data, a group size of 28 subjects has over 95% power to achieve a difference in TG of 243.5 mg/dL (median 1: 745.5 mg/dL; median 2: 502.0 mg/dL; estimated SD: 270 mg/dL). Considering an estimated dropout rate of 5–10%, at least 30 subjects per group were to be recruited for the MoKaRi II study.

The participants were randomly assigned to either the intervention or the control group within each study arm based on a simple randomization list, which was generated with the statistical software R version 3.5.1 (The R Foundation for Statistical Computing, Vienna, Austria).

All statistical tests were performed using the statistical software IBM SPSS statistics version 29.0.0.0 (241) (IBM Germany, Ehningen, Germany). Whether the data followed a normal distribution was determined using the Shapiro–Wilk test. Differences within groups while comparing each point in time were assessed using ANOVA for repeated measurements for normally distributed variables or the Friedmann test if they were not normally distributed. As a post-hoc test, Fisher’s least significant difference test was used, and all calculated *p*-values were adjusted manually using the Benjamini–Hochberg procedure [37]. Differences between each intervention group and their corresponding control group, as well as differences between both intervention groups (including all tests comparing changes from baseline between groups), were assessed using independent sample t-test for normally distributed data or Mann–Whitney-U test for not normally distributed data. Only data from subjects who attended every study appointment were included for all tests, except those where the change from baseline calculations were performed. For the change from baseline tests, subjects only had to be present at the relevant time points (baseline, week 10, week 20). Correlations were calculated using Pearson’s correlation if the required criteria (metric data, linear relationship, no outliers; bivariate normal distribution assumed based on central limit theorem) were met, or alternatively using Spearman’s rank correlation.

## 3. Results

### 3.1. Subjects

Sorted by group, 34, 33, 37, and 32 subjects started the study, whereas only 30, 33, 30, and 31 subjects completed the study to the extent that they could at least be included in one statistical analysis (Figure 1). This dropout rate of 8.2% is within the expected range of the power calculation.

Table 1 and Table 2 present the basic characteristics of each study arm. Only subjects who completed the intervention period of the study were included in these calculations. In both study arms, both age and BMI were within the range of the defined inclusion criteria. Median blood glucose (5.8 (5.4, 6.4) mmol/L) met the inclusion criterion in the prediabetes study arm, whereas median TG (1.6 (1.2, 2.0) mmol/L) was slightly below the defined cutoff in the hypertriglyceridemia study arm (Table 1). About 70% women were included in study arm 1 and about 77% in study arm 2. A comparison of both intervention groups with their corresponding control group shows a balanced distribution. The age of the subjects in each study arm, and between both intervention groups, did not differ significantly from each other (Table 2).

### 3.2. Nutrient Intake

The results of the 5-day dietary self-report before baseline assessment mostly showed a comparable intake of energy, macronutrients, vitamins, minerals, and trace elements in both study arms and between both intervention groups. In detail, there were only differences in the consumption of alcohol (1.9 (0.2, 13.5) vs. 10.9 (3.9, 23.2) g/d) between the HTGI and hypertriglyceridemia control (HTGC) groups and of sugar (90 (73, 125) vs. 117 (95, 128) g/d), glucose (15.4 (12.2, 21.7) vs. 20.9 (16.4, 25.2) g/d), and fructose (18.9 (13.9, 25.6) vs. 26.6 (21.0, 31.9) g/d) between the HTGI and PDI groups (*p* < 0.05) (Table S2). Compared to the reference values of the DGE (adults aged 51–64 years), the average daily intake in all groups for saturated fatty acids (SFA), cholesterol, phosphorus, sodium, and chloride was higher, and the intake of fiber, PUFA, vitamin A, calcium, and potassium was lower than recommended daily amounts (Table S2). In comparison to the criteria defined for the menu plans, there were differences in both the HTGI and PDI groups, in which higher amounts of sucrose + glucose + fructose (15 and 17 percent of daily energy intake (en%)), SFA (16 en% in both groups), and cholesterol (363 and 369 mg/d) as well as lower amounts of fiber (24 and 28 g/d), PUFA (6 and 5 en%), and EPA + DHA (0.3 and 0.4 g/d) were consumed before the start of the intervention (Figure 3, Table S2).

### 3.3. Cardiovascular and Diabetic Risk Factors

The 10-week dietary intervention resulted in decreases of total low-density lipoprotein (LDL), high-density lipoprotein (HDL), and non-HDL cholesterol, TG, fasting glucose, HbA1c, insulin, connecting peptide (C-peptide), homeostatic model assessment for insulin resistance (HOMA-IR), triglyceride glucose (TyG) index, fatty liver index (FLI), systolic blood pressure, body weight, BMI, body fat (kg and %), lean body mass (kg), total body water (L), and waist circumference in both intervention groups (*p* < 0.05). In addition, a reduction in high-sensitivity c-reactive protein (CRP) and visceral adipose tissue (VAT) was observed in the HTGI group, and in diastolic blood pressure in the PDI group (*p* < 0.05). After 10 additional weeks of follow-up, total and LDL cholesterol, C-peptide, FLI, high-sensitivity CRP, and systolic blood pressure were lower than at baseline in the HTGI group (*p* < 0.05). In the PDI group, this was the case for C-peptide, FLI, non-HDL cholesterol, and HbA1c (*p* < 0.05). Anthropometric parameters (body weight, BMI, body fat (kg and %), lean body mass (kg), total body water (L), and waist circumference) were also lower in both intervention groups at follow-up compared to their baseline values (*p* < 0.001) (Table 3, Table 4 and Table 5).

The mentioned reductions (%^A→F^) in the biochemical and anthropometric parameters were more pronounced in the intervention groups than in their respective control groups (*p* < 0.05), except for the reduction of HDL cholesterol, fasting glucose, and systolic blood pressure in the PDI group and for diastolic blood pressure in either intervention group. Regarding total, LDL and non-HDL cholesterol, HbA1c, C-peptide, FLI, and anthropometric parameters (body weight, BMI, body fat (kg and %), total body water (L), lean body mass (kg), waist circumference), this was also the case at follow-up (*p* < 0.05). Moreover, the reduction of systolic blood pressure (*p* = 0.014) and VAT (*p* < 0.001) in the HTGI group and of TG, insulin, HOMA-IR, and TyG index (*p* < 0.05) in the PDI group were greater at follow-up. At the end of the intervention, the HTGI group showed lower total, LDL, non-HDL cholesterol, TG, fasting glucose, C-peptide concentrations, and a lower TyG index than the HTGC group (*p* < 0.05). In addition, the same applies to FLI, body weight, waist circumference, and VAT after the intervention and at follow-up (*p* < 0.05). The PDI group had reduced total cholesterol, HDL cholesterol, insulin concentrations, and HOMA-IR after 10 weeks of dietary intervention compared to the prediabetes control (PDC) group (*p* < 0.05) (Table 3, Table 4 and Table 5).

Regarding baseline values, the PDI group had higher TG concentrations, TyG index, FLI, body weight, waist circumference, and lower HDL cholesterol concentrations than the PDC group (*p* < 0.05) (Table 3 and Table 4).

When comparing the two intervention groups, a higher reduction in FLI was observed in the HTGI than in the PDI group (*p* = 0.035). In addition, higher total cholesterol concentrations at baseline (*p* = 0.025) and a higher pulse rate at the end of the intervention and at follow-up (*p* < 0.05) were detected in the HTGI group. A comparison of bioelectrical impedance analysis data among the two intervention groups was not performed due to different instruments used for measurement (Table 3).

Correlation analyses between absolute changes (baseline vs. end of the intervention) in body weight and in LDL cholesterol, TG, fasting glucose, HOMA-IR, or HbA1c showed no correlations in the HTGI group (r = −0.002, *p* = 0.990; r = 0.185, *p* = 0.336; r = 0.311, *p* = 0.101; r = 0.204, *p* = 0.287; r = 0.013, *p* = 0.945, respectively) nor in the PDI group (r = 0.233, *p* = 0.215; r = 0.223, *p* = 0.237; r = 0.040, *p* = 0.833; r = 0.107, *p* = 0.574; r = 0.099, *p* = 0.604, respectively).

**Figure 4:**
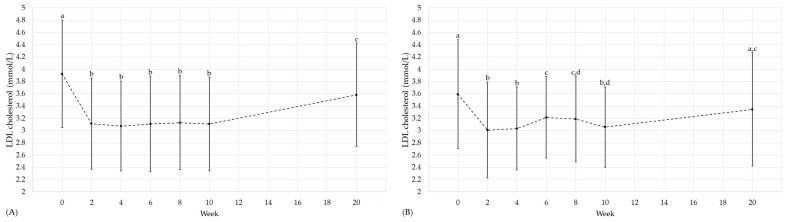
LDL cholesterol (mmol/L) over the course of the study in the HTGI (**A**) and PDI (**B**) groups. Showing data at baseline (week 0), after weeks 2, 4, 6, 8 and 10 and the follow-up (week 20). Data expressed as mean (±SD) according to the statistical test that was performed. Data points in time without a common letter are significantly different, *p* < 0.05. (**A**) Data per time: 0: 3.9 (±0.9); 2: 3.1 (±0.7); 4: 3.1 (±0.7); 6: 3.1 (±0.8); 8: 3.1 (±0.8); 10: 3.1 (±0.8); 20: 3.6 (±0.8). (**B**) Data per time: 0: 3.6 (±0.9); 2: 3.0 (±0.8); 4: 3.0 (±0.7); 6: 3.2 (±0.7); 8: 3.2 (±0.7); 10: 3.1 (±0.7); 20: 3.3 (±0.9). Abbreviations: LDL, low-density lipoprotein.

**Figure 5:**
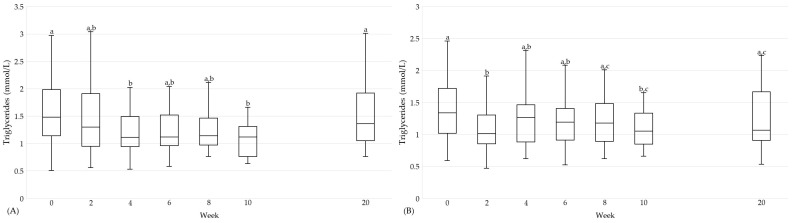
Triglycerides (mmol/L) over the course of the study in the HTGI (**A**) and PDI (**B**) groups. Showing data at baseline (week 0), after weeks 2, 4, 6, 8, and 10 and the follow-up (week 20). Data expressed as median (25th, 75th percentile) according to the statistical test that was performed. Data points in time without a common letter are significantly different, *p* < 0.05. (**A**) Data per time: 0: 1.5 (1.1, 2.0); 2: 1.3 (1.0, 1.9); 4: 1.1 (0.9, 1.5); 6: 1.1 (1.0, 1.5); 8: 1.1 (1.0, 1.5); 10: 1.1 (0.8, 1.3); 20: 1.4 (1.1, 1.9). (**B**) Data per time: 0: 1.3 (1.0, 1.7); 2: 1.0 (0.9, 1.3); 4: 1.3 (0.9, 1.5); 6: 1.2 (0.9, 1.4); 8: 1.2 (0.9, 1.5); 10: 1.1 (0.8, 1.3); 20: 1.1 (0.9, 1.7).

**Figure 6:**
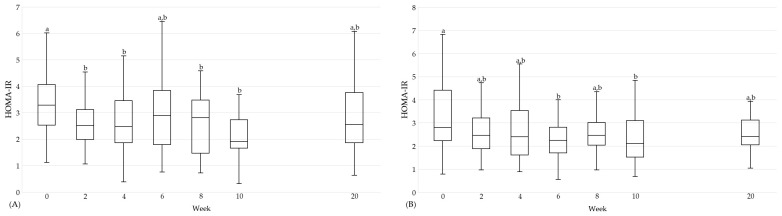
HOMA-IR over the course of the study in the HTGI (**A**) and PDI (**B**) groups. Showing data at baseline (week 0), after weeks 2, 4, 6, 8, and 10 and the follow-up (week 20). Data expressed as median (25th, 75th percentile) according to the statistical test that was performed. Data points in time without a common letter are significantly different, *p* < 0.05. (**A**) Data per time: 0: 3.3 (2.5, 4.1); 2: 2.5 (2.0, 3.1); 4: 2.5 (1.9, 3.5); 6: 2.9 (1.8, 3.8); 8: 2.8 (1.5, 3.5); 10: 1.9 (1.7, 2.7); 20: 2.5 (1.9, 3.8). (**B**) Data per time: 0: 2.8 (2.2, 4.4); 2: 2.5 (1.9, 3.2); 4: 2.4 (1.6, 3.5); 6: 2.2 (1.7, 2.8); 8: 2.5 (2.0, 3.0); 10: 2.1 (1.5, 3.1); 20: 2.4 (2.1, 3.1). Abbreviations: HOMA-IR, Homeostatic Model Assessment for Insulin Resistance.

It can be highlighted that positive effects on cardiovascular and diabetic risk factors did not only occur at the end of the intervention. In the following, this is shown for selected parameters (Figure 4, Figure 5 and Figure 6). In the HTGI group, LDL cholesterol and HOMA-IR were already reduced after two weeks (*p* < 0.05; Figure 4 and Figure 6). With regard to TG, a reduction was observed for the first time after four weeks of intervention (*p* < 0.05; Figure 5). For LDL cholesterol, the effect remained throughout the intervention (*p* < 0.05), whereas for TG and HOMA-IR there was a higher variability (Figure 4, Figure 5 and Figure 6). In the PDI group, a decrease was observed in LDL cholesterol for the first time after two weeks, which was maintained for the duration of the intervention compared to baseline (*p* < 0.05; Figure 4). For TG and HOMA-IR, a reduction was observed for the first time after two and six weeks, respectively (*p* < 0.05; Figure 5 and Figure 6).

The baseline data of both intervention groups, compared to the lowest value of each subject observed within the intervention period, showed inter alia reductions in body weight, LDL cholesterol, TG, and HOMA-IR (*p* < 0.001), which was more pronounced for LDL cholesterol in the HTGI than in the PDI group (absolute and percentage change from baseline, *p* = 0.004). In detail, body weight fell by 8% and 7%, LDL cholesterol by 27% and 20%, TG by 30% and 28%, and HOMA-IR by 47% and 39% in the HTGI and PDI groups, respectively (Table 6).

The lowest values in body weight were mainly observed after week 10 (HTGI: 76%; PDI: 70%). For LDL cholesterol, the lowest values were mainly observed after 2 (31%) and 10 (28%) weeks in the HTGI group, whereas this applied to weeks 2 (33%) and 4 (27%) in the PDI group. For TG, the lowest value in the PDI group was mainly observed after week 2 (40%), whereas in the HTGI group this was equally distributed after weeks 4 (31%) and 10 (31%). The lowest values of HOMA-IR were most frequently observed in the HTGI group after weeks 4 (28%) and 10 (38%), whereas there was a primarily even distribution in the PDI group (Table 7).

### 3.4. Nutrient Status

A decrease in micronutrients, specifically vitamin B_12_ and ferritin concentrations, was observed in all groups after the intervention (*p* < 0.01). The decrease in vitamin B_12_ was higher in the PDI than in the PDC group (*p* = 0.036), resulting in lower concentrations at the end of the intervention period (*p* = 0.005). Ferritin concentrations were also lower at follow-up in all groups (*p* < 0.05), with the change from baseline being more pronounced in the HTGC than in the HTGI group (*p* = 0.038). Transferrin concentrations decreased during the intervention in the HTGI group (*p* = 0.007), with the reduction (%^A→F^) also being greater than in the HTGC group (*p* = 0.023). No significant changes were observed in transferrin saturation. However, the PDC group showed higher values than the PDI group both at baseline and after the intervention (*p* < 0.05). A decrease in vitamin E concentration was detected during the intervention in the HTGI group alone (*p* < 0.001), whereas the percentage reduction was greater in both the HTGI and PDI groups compared to the HTGC and PDC groups (*p* < 0.01), respectively. These changes resulted in lower concentrations at the end of the intervention (*p* < 0.05). The PDC group’s concentration increased after both 10 and 20 weeks (*p* < 0.05). Furthermore, the vitamin B_1_ concentrations in the HTGI and HTGC groups (*p* < 0.001) and the vitamin A concentrations in the PDI group (*p* = 0.038) decreased at the end of the intervention. In contrast, an increase in vitamin A level was observed in the PDC group (*p* = 0.033). The change from baseline (%^A→F^) of vitamin A concentration differed between the PDI and the PDC group (*p* = 0.001). At the end of the intervention, vitamin B_6_ concentrations increased in the HTGI, PDI, and PDC groups, vitamin D concentrations in the HTGC, PDI, and PDC groups, and folic acid concentration in the PDI group (*p* < 0.05). The changes from baseline of folic acid concentrations (%^A→F^ and %^A→G^) differed between the PDI and the PDC group (*p* < 0.05). The vitamin D concentration in the HTGI group was reduced at follow-up compared to baseline and the end of the intervention (*p* < 0.05). The baseline vitamin D concentration was higher in the HTGI than in the HTGC group (*p* = 0.032) (Table S3).

When comparing the two intervention groups, higher vitamin E, vitamin D, and folic acid concentrations were observed in the HTGI group at baseline (*p* < 0.05). Vitamin D and B_6_ concentrations were higher after the intervention (*p* < 0.05). The increased folic acid concentration in the PDI group at the end of the intervention and the decreased vitamin D concentration at follow-up in the HTGI group were also evident when comparing the changes from baseline between the intervention groups (*p* < 0.05) (Table S3).

Changes in urinary parameters were observed in creatinine, sodium, chloride, and selenium throughout the study (*p* < 0.05). Creatinine concentrations were lower after 10 weeks of intervention (*p* = 0.014), and selenium concentrations were lower at both week 10 and 20 in the HTGI group than at baseline (*p* = 0.032). The creatinine concentration was lower in the HTGI than in the HTGC group at the end of the intervention (*p* = 0.024). Moreover, the changes from baseline (%^A→F^ and %^A→G^) differed between the two groups (*p* < 0.05). The reduction in selenium concentration in the HTGI group resulted in a difference in change from baseline (%^A→F^) between the HTGI and the HTGC group (*p* = 0.042). When comparing the PDI and PDC groups, lower selenium levels at baseline and a higher increase in change from baseline (%^A→G^) were observed in the PDI group (*p* < 0.05). Furthermore, zinc concentrations were higher in the PDI than in the PDC group at both the beginning and end of the intervention (*p* < 0.05). Lower magnesium concentrations were observed in the HTGI than in the HTGC group after the intervention (*p* = 0.029) (Table S4).

When comparing the two intervention groups, the HTGI group had higher selenium levels at baseline (*p* = 0.004), and the PDI group had higher magnesium and zinc levels at the end of the intervention (*p* < 0.01). Chloride, magnesium, sodium, and selenium concentrations showed a negative trend in the HTGI group (exception: decrease in selenium (*p* = 0.032)) and a positive trend in the PDI group, which resulted in significant differences in change from baseline (%^A→F^) and for selenium additionally in change from baseline (%^A→G^) (Table S4).

### 3.5. Fatty Acid Distribution in Erythrocyte Lipids

An essential component of the nutritional intervention was the supplementation of fish oil in the HTGI group as well as the consumption of *α*-linolenic acid (ALA)-rich foods (plant oils, nuts, seeds) and high-fat sea fish in the PDI group. The characteristic fatty acid profile of these oils or foods is reflected in the erythrocyte fatty acids.

In the HTGI group, EPA increased by 219 (120, 270)%, docosapentaenoic acid (DPA) by 53 (15, 95)%, DHA by 37 (1, 66)%, *n*-3 index by 61 (38, 100)%, and total *n*-3 PUFA by 57 (24, 88)% during the 10-week intervention (*p* < 0.001). After another ten weeks of follow-up, these fatty acids were 62 (29, 150)%, 35 (19, 59)%, 26 (11, 40)%, 36 (16, 59)%, and 35 (15, 52)% higher than at baseline, respectively (*p* < 0.01). The ALA concentration remained unchanged. Consequently, the EPA/ALA, DPA/ALA, and DHA/ALA ratios also increased (after 10 weeks: *p* < 0.01, after 20 weeks: *p* < 0.05). The percentage change from baseline and the concentrations of the previously mentioned *n*-3 fatty acids and the values of the ratios were, with two exceptions (change from baseline (%^A→G^) of DPA/ALA and DHA/ALA ratios), higher than in the HTGC group after 10 and 20 weeks (*p* < 0.05) (Table 8).

In the PDI group, there was a 67 (±67)% increase in ALA, which was higher than in the PDC group (*p* < 0.001). At follow-up, the increase was 27 (−14, 50)% (*p* = 0.021). Also, the concentration was higher in the PDI than in the PDC group after 10 and 20 weeks (*p* < 0.01). Furthermore, the concentration of *n*-3 PUFA increased by 12% in the PDI group at the end of the intervention and by 10% at follow-up compared to baseline (*p* < 0.05). These values were higher at the end of the intervention than in the PDC group (*p* < 0.05). The increase in long-chain *n*-3 PUFA EPA, DPA, DHA, and *n*-3 index only reached significance at follow-up. These increased by a median of 20%, 16%, 7%, and 6%, respectively (*p* < 0.05). Differences in concentrations compared to the PDC group were not detected. The EPA/ALA, DPA/ALA, and DHA/ALA ratios decreased significantly after the 10-week intervention and showed lower values than in the PDC group (*p* < 0.05). The DPA/ALA and DHA/ALA ratios were also lower in the PDI than in the PDC group at follow-up (*p* < 0.05). At 10 weeks, the percentage increase in DHA and *n*-3 index and the percentage decrease of EPA/ALA, DPA/ALA, and DHA/ALA ratio was higher in the PDI than in the PDC group (*p* < 0.05). This also was true at follow-up for the EPA/ALA ratio (*p* = 0.048) (Table 8).

By comparing both intervention groups, a significantly higher increase and higher concentration at the end of the intervention was observed in ALA in the PDI group and in EPA, DPA, DHA, total *n*-3 PUFA, *n*-3 index, and EPA/ALA, DPA/ALA, and DHA/ALA ratios in the HTGI group (*p* < 0.01, exception: DHA %^A→F^ *p* = 0.126). The same effects were also observed at follow-up (*p* < 0.01, exception: DHA %^A→G^ *p* = 0.01) (Table 8).

A reduction of the *n*-6 fatty acids dihomo-*γ*-linolenic acid (DGLA), arachidonic acid (ARA), and total *n*-6 PUFA concentrations was observed in the HTGI group after 10 weeks (30%, 12%, 15%, respectively) and 20 weeks (14%, 7%, 6%, respectively) (*p* < 0.01, exception: DGLA %^A→G^ *p* = 0.011). Concentrations were lower in the HTGI group at the end of the intervention and at follow-up compared to the HTGC group (*p* < 0.05, exception: DGLA at follow-up). Linoleic acid (LA) was lower at the end of intervention, and C22:4*n*6 was lower at follow-up compared to baseline and to the HTGC group (*p* < 0.05). The percentage reduction in LA and total *n*-6 PUFA was more pronounced in the HTGI than in the HTGC group at the end of the intervention and additionally for DGLA and C22:4*n*6 at follow-up (*p* < 0.05). A higher reduction in the ARA/LA ratio was observed at the end of the intervention in the HTGC group than in the HTGI group (*p* < 0.05) (Table 8).

In the HTGI group, the increased concentrations of *n*-3 fatty acids and the decreased concentrations of *n*-6 fatty acids are reflected in lower *n*-6/*n*-3, ARA/EPA, and ARA/DHA ratios after the intervention and at follow-up compared to baseline and to the HTGC group (*p* < 0.01). The percentage reduction in these ratios was also higher (*p* < 0.01) (Table 8).

A reduction in the *n*-6 fatty acid DGLA was observed in the PDI and PDC groups after 10 weeks compared to baseline (*p* < 0.05). In contrast, LA concentration increased in the PDI group after 10 weeks compared to baseline (*p* = 0.013). The ARA/LA ratio was lower in the PDI group at the end of intervention and at follow-up and in the PDC group at the end of intervention compared to baseline (*p* < 0.05) (Table 8).

In the PDI group, a reduction of the LA/ALA ratio was observed at the end of the intervention and a reduction of the *n*-6/*n*-3 and ARA/DHA ratio additionally at the follow-up (*p* < 0.05). Therefore, the reduction of the LA/ALA ratio (after 10 weeks) was higher than in the PDC group (*p* = 0.003). Lower values compared to the PDC group were observed in the LA/ALA ratio after the intervention and at follow-up (*p* < 0.01) and in the ARA/DHA ratio at the end of the intervention (*p* = 0.035) (Table 8).

When comparing both intervention groups, *n*-6 PUFA concentrations and ratios of *n*-6 to *n*-3 PUFAs tended to fall more in the HTGI than in the PDI group. A significantly higher reduction and a significantly lower value at the end of the intervention was observed in LA, DGLA, and total *n*-6 PUFA concentration and in the *n*-6/*n*-3, ARA/EPA, and ARA/DHA ratios. This was true at follow-up for ARA, C22:4*n*6 and total *n*-6 PUFA and for the ratios mentioned before (*n*-6/*n*-3 PUFA, ARA/EPA, and ARA/DHA) (*p* < 0.05). The percentage change from baseline in DGLA also was more pronounced at follow-up (*p* = 0.001) in the HTGI group, but did not differ in the concentration compared to the PDI group. In contrast, a higher reduction in the LA/ALA ratio was observed in the PDI group at follow-up (*p* = 0.046). The values were lower than in the HTGI group at the end of the intervention (*p* = 0.026) and at follow-up (*p* = 0.008). Moreover, after 10 weeks, the ARA/LA ratio reduced further and reached a lower value in the PDI group (*p* < 0.001) (Table 8).

In addition to the changes in *n*-3 and *n*-6 fatty acids, a lower C14:0, C15:0, and trans-fatty acid (TFA) concentration were measured in the HTGI group at the end of the intervention compared to baseline (*p* < 0.05). The reduction of C15:0 was more pronounced after the intervention period, and the reduction of C14:0 was more pronounced after the intervention period and at follow-up in the HTGI than in the HTGC group (*p* < 0.05). At the end of the intervention, the HTGI group had a lower C14:0 concentration than the HTGC group (*p* < 0.001) (Table 8).

In the PDI group, a decrease in C14:0 and conjugated linoleic acid-c9,t11/t8,c10 was observed after the intervention compared to baseline (*p* < 0.01). The reduction in C14:0 was higher than in the PDC group both at the end of intervention (*p* < 0.001) and at follow-up (*p* = 0.013). After the intervention, a lower C14:0 concentration was observed in the PDI compared to the PDC group (*p* < 0.001). Even though C18:0 did not drop significantly in the PDI group, the concentration was lower than in the PDC group after 10 weeks (*p* = 0.018). C16:0, C17:0, C18:1c9, total monounsaturated fatty acids (MUFA) concentration and C18:1c9/C18:0 ratio increased during the intervention in the PDI group (*p* < 0.01, exception: C16:0 *p* = 0.028). A higher value at the end of the intervention was observed in the C18:1c9/C18:0 ratio compared to the PDC group (*p* = 0.005). At follow-up, in the PDI group, C17:0 levels remained higher compared to baseline (*p* = 0.003), whereas C16:0 levels were lower (*p* = 0.015). There was also an increase in C17:0, C18:1c9, and total MUFA levels at the end of the intervention in the PDC group (*p* < 0.01, exception: total MUFA *p* = 0.043) (Table 8).

When comparing the two intervention groups, a higher reduction in C15:0 and TFA was observed in the HTGI group after the intervention, and a greater increase in C18:1c9, total MUFA, and C18:1c9/C18:0 ratio in the PDI group (*p* < 0.01). In addition, a non-significant increase in C18:0 in the HTGI group differed from a non-significant decrease in the PDI group (*p* = 0.016). Accordingly, C15:0, C18:1c9, total MUFA concentrations and C18:1c9/C18:0 ratio were lower and C18:0 and total SFA concentration were higher in the HTGI group (*p* < 0.05). TFA concentration was higher at baseline, and C16:0 concentration was higher at baseline and follow-up in the HTGI group (*p* < 0.05) (Table 8).

## 4. Discussion

### 4.1. Effects on Cardiovascular and Diabetic Risk Factors

The 10-week nutritional intervention led to equal and significant improvements in cardiovascular and diabetic risk markers in both intervention groups (exception: greater reduction in FLI in the HTGI group). In particular, body weight was reduced by 7% and 8%, LDL cholesterol by 20% and 13%, TG by 18% and 13%, glucose by 4% and 5%, HbA1c by 3% and 5%, and HOMA-IR by 32% and 26%, respectively (*p* < 0.05). These beneficial changes were sustained for some parameters after 10 weeks of follow-up. These improvements mostly differ from the effects observed in the corresponding control groups.

Assuming that each subject’s compliance fluctuated throughout the study, we analyzed the lowest values observed for each subject during the intervention period to visualize the interventions’ maximum potential. The results showed a decrease in LDL cholesterol by an average of 27% and 20%, TG by 30% and 28%, and HOMA-IR by 47% and 39% in the HTGI and PDI groups, respectively. These markedly more pronounced reductions compared to the average reductions at the end of the intervention underline the high relevance of continuous and long-term implementation of the concepts.

A possible explanation for the reduction of TG might be the role of CHO [38]. CHO inhibit the β-oxidation of fatty acids in the liver since CHO are preferentially used for energy production [39]. Therefore, more fatty acids are available for VLDL synthesis and release into the bloodstream [39]. Furthermore, increased CHO intake results in increased de novo lipogenesis, which also contributes to the increase in TG [40]. Compared to the 5-day dietary self-report recorded before the study, the CHO intake was reduced from 246 to 183 g/d in the prediabetes concept. We suspect that this contributed in part to the TG reduction observed in the PDI group. Furthermore, studies have also shown effects of optimized CHO quality on TG, which is characterized by an increased fiber intake and reduced consumption of refined CHO [41,42,43]. Accordingly, the effects observed in the HGTI and PDI group could be partly attributable to the quality of the CHO consumed.

Previous studies confirmed the beneficial effect of a CHO restricted diet on TG concentrations. Archer et al. (2003) and Rajaie et al. (2014) showed that in both men with normal TG concentrations and women with elevated TG concentrations, a low CHO diet (about 46 en% CHO, 16 en% protein) resulted in significant reductions in TG compared to a high-CHO diet (about 59 en% CHO, 16 en% protein) [44,45]. Archer et al. detected 17% and Rajaie et al. a 0.35 mmol/L decrease in TG levels [44,45]. Volk et al. (2014) also showed in their study that a low-CHO diet (29 or 40 en% CHO, 20% protein) compared to a high-CHO diet (55 en% CHO, 20 en% protein) resulted in significantly lower TG concentrations of about 0.4 or 0.36 mmol/L in subjects with normal TG concentrations [46]. The implementation of the nutritional concepts was associated with weight loss, which could also be responsible for the observed TG-lowering effect. However, even independent of weight loss, a low-CHO diet (43–48 en% CHO) has been shown to result in significantly lower TG concentrations compared with a high-CHO diet (55–65 en% CHO) in subjects with normal TG concentrations [47,48,49]. The meta-analyses of Schwingshackl and Hoffmann (2013) and Cao et al. (2009) also confirmed that a high-fat diet (>30 en% fat), which consequently has a lower CHO proportion, leads to significantly lower TG concentrations compared to a low-fat diet (≤30 en% fat) regardless of weight loss [50,51]. Consistent with these data, no correlation between weight loss and the reduction in TG was found in the present study (HTGI: r 0.221, *p* = 0.240, PDI: r 0.223, *p* = 0.237). In contrast, Thorning et al. (2015) and Zheng et al. (2008) found no effect on TG concentrations due to low-CHO diets (around 49 en% CHO, 15 en% protein, 36 en% fat) vs. high-CHO diets (around 63 en% CHO, 15 en% protein, 22 en% fat) in subjects with normal TG concentrations and stable body weight [52,53].

Regarding total, LDL, non-HDL, and HDL cholesterol, a diet with lower CHO did not lead to significant differences compared to a diet with a higher CHO proportion [45,46,49,53]. However, Shin et al. (2007) found higher total, LDL, and HDL cholesterol levels in participants with a low-CHO diet, whereas Archer et al. (2003) and Thorning et al. (2015) only found higher HDL cholesterol levels [44,47,52]. According to the meta-analyses of Schwingshackl and Hoffmann (2013) and Cao et al. (2009), low-CHO diets lead to higher HDL cholesterol concentrations, whereas no differences or even elevated values were observed in total and LDL cholesterol [50,51].

Since CHO cause an increase in blood glucose levels and, consequently, insulin levels, it can be assumed that the reduced CHO intake in the PDI group is responsible for the decrease of each marker of glucose metabolism. Studies comparing a diet with reduced CHO intake (30–45 en%) with a diet high in CHO intake (45–60 en%) often reveal improvements in glucose metabolism parameters within low-CHO groups but usually only significant differences in HbA1c values compared to high-CHO groups [54,55,56]. Elhayany et al. (2010) and Skytte et al. (2019) observed a stronger reduction in HbA1c as a result of reduced CHO intake in patients with DMT2, whereas a stronger reduction in blood glucose was only observed by Skytte et al. [54,55]. There were no significant differences in HOMA-IR, insulin, and C-peptide concentrations between the diets [54,55]. Brunerova et al. (2007) did not observe significant differences in glucose, insulin, C-peptide, HOMA-IR, and HbA1c values between the two diets in subjects with and without DMT2 [56]. All three studies showed no significant differences in weight loss between the diets [54,55,56]. In their meta-analysis, Sainsbury et al. (2018) did not find a significant difference in HbA1c in patients with DMT2 (one study with people with type 1 diabetes mellitus included) after 3, 6, or 12 months on a diet with 33–45 en% CHO compared to a diet with >45 en% CHO. Reducing CHO to below 26 en% resulted in a significantly greater reduction in HbA1c values at 3 and 6 months compared with a CHO intake of >45 en%. However, at 3 months these low-CHO diets (<26 en% CHO) led to greater weight loss than the high-CHO diets (>45 en% CHO). Sensitivity analyses were conducted to test the relevance of weight loss on HbA1c values. These excluded studies with significantly greater weight loss due to low-CHO diets. At 3 months, there were no longer significant differences in HbA1c change between the low- and high-CHO diets [26]. These results suggest that weight loss can contribute to the improvement of glucose metabolism parameters. This was confirmed by Beavers et al. (2013), who observed that weight loss achieved by reduced caloric intake (without a specific macronutrient reduction, with or without physical activity) was associated with reductions of glucose, insulin, and HOMA-IR [57].

In addition to a reduced CHO intake, the intake of EPA and DHA influences TG concentrations. A high-dose intake of 3–4 g EPA and/or DHA daily can lead to a 15–26% reduction in TG concentrations [58,59,60,61,62]. This effect occurs independently of changes in the diet and body weight and in individuals with both normal (<1.7 mmol/L) and elevated TG concentrations (≥1.7 mmol/L) [58,59,60,61,62]. In this context, the higher the initial TG concentrations are, the greater the reduction [36,58]. The following mechanisms are associated with the TG-lowering effect of EPA and DHA: EPA and DHA reduce hepatic TG synthesis by inhibiting enzymes involved in fatty acid and TG synthesis, increase β-oxidation by interacting with peroxisome proliferator-activated receptor-α, and improve clearance of VLDL and chylomicrons by increasing lipoprotein lipase activity [63].

In the present study, the subjects in the HGTI group supplemented 3.5 g/d EPA + DHA, which is a possible explanation for the observed reduction in TG concentrations. A comparison of both intervention groups shows that the supplementation of EPA + DHA or a reduced CHO intake led to comparable effects on TG concentrations (−18% vs. −13%, n.s.). In line with the literature, significant effects can also be achieved with TG concentrations < 1.7 mmol/L.

In addition, the implementation of the menu plans results in a comparably low intake of SFA and cholesterol as well as high fiber intake. This may explain the beneficial effects on total, LDL, and non-HDL cholesterol. SFA and cholesterol increase LDL concentrations by inhibiting LDL receptor activity and enhancing apolipoprotein B-containing lipoprotein production [64]. In this context, the shorter-chain SFA C12:0, C14:0, and C16:0 have a more substantial LDL-increasing effect than C18:0 [64], with C:16:0 being the most common SFA in many diets in terms of quantity [65]. If energy derived from SFA is replaced by *n*-6 PUFA or MUFA, LDL cholesterol decreases [30]. Here, the LDL-lowering effect of *n*-6 PUFA is stronger than that of MUFA [30]. *n*-3 PUFA have no hypocholesterolemic effect, probably because they enhance the conversion of VLDL to LDL [30,66]. However, the effect of SFA and cholesterol on LDL concentrations varies interindividually because it is determined by apolipoprotein E and Niemann-Pick C1-Like 1 polymorphism, among other factors [64,67,68].

Dietary fiber, especially soluble fiber, and whole grain products, which have higher fiber contents than refined flour products, lower total and LDL cholesterol, glucose, and insulin and are thus considered to have health-promoting effects [69,70,71,72]. Possible mechanisms for these effects include increased excretion of bile acids and cholesterol and slowed glucose absorption due to the viscous properties of soluble fiber [70,72]. The reduced reabsorption of bile acids leads to an increased cholesterol intake from the blood into the liver, as this is required to form new bile acids [70].

The meta-analyses of Sun et al. (2015) and Schoeneck and Iggman (2021) confirmed the beneficial effect on LDL cholesterol levels when foods rich in SFA were replaced with foods low in SFA [73,74]. The beneficial effect of soluble dietary fiber on LDL cholesterol and parameters of glucose metabolism has been also described in several meta-analyses [74,75,76].

However, in addition to the effects of specific nutrients on blood lipids, weight loss is also associated with reductions in total and LDL cholesterol [57,77,78,79], whereby no correlation between weight loss and reduction in LDL cholesterol was observed in the present study (HTGI: r 0.087, *p* = 0.649; PDI: r 0.285, *p* = 0.169).

The TyG index is a reliable and convenient surrogate for insulin resistance [80,81]. It is positively associated with cardiometabolic risk factors and, therefore, with the risk of developing coronary heart disease and diabetes [80,82,83]. Reference values for the TyG index in relation to cardiovascular or diabetes risk are currently not available. Previous studies divide the TyG index into tertiles or quartiles based on the given cohort and established an association with CVD [80,83,84,85]. Due to the lack of reference values, we can only conclude that both interventions significantly reduced the TyG index.

The FLI helps to identify hepatic steatosis [86,87], whereby subjects having an FLI ≥ 60 are categorized as having hepatic steatosis and FLI < 30 as not [87]. The FLI is positively associated with the development of DMT2 and the Framingham 10-year CVD risk [88,89,90]. Seo et al. (2022) observed in their study that individuals without DMT2 and an FLI ≥ 60 have a 2.98 times higher risk of developing DMT2 than those with an FLI < 30 [88]. In the IT-DIAB study, 40.7% of patients with impaired fasting glucose (≥110 and <126 mg/dL) and an FLI of ≥ 60 developed DMT2 within 5 years, compared to 19.5% of patients with an FLI < 30 [89]. In the study by Chung et al. (2016), the odds ratio for a Framingham 10-year CVD risk ≥ 10% was 2.56 times higher in individuals with an FLI ≥ 60 than in individuals with an FLI < 30 [90]. At baseline, the HTGI and PDI groups had a median FLI above 60 (70.0 (45.8, 89.6) and 71.6 (47.8, 91.4), respectively). The intervention significantly reduced the FLI to 38.5 (16.3, 63.0; −38.3%) and 53.4 (25.8, 73.1; −25.3%), respectively, which is below the critical value described in previous findings but may contribute to a reduced cardiovascular risk.

In the long term, the observed effects on various cardiovascular and diabetic risk factors could have a combined effect in the prevention of associated diseases if maintained over time, as also shown by Gæde et al. (2003) in an intervention study on CVD in people with DMT2 [91].

### 4.2. Effects on Nutrient Status

The menu plans used in both intervention groups ensured that the intake of vitamins, minerals, and trace elements was assumed to be according to the subjects’ micronutrient requirements following the reference values for nutrient intake of the DGE. Since all study groups mostly covered the micronutrient requirements (except for vitamin A, calcium, and potassium) based on the data from the 5-day dietary protocols concerning the DGE reference values, the menu plans were not expected to significantly improve micronutrient supply.

The significant decrease in the HTGI group and non-significant decrease in the PDI group of vitamin E is presumably attributable to the observed reduction in lipoprotein concentrations [92]. The increased PUFA intake in the HTGI group as a result of fish oil supplementation could have further increased this effect [93].

In our study, the supplementation of fish oil in the HTGI group led to a significant increase in EPA, DPA, DHA, *n*-3 index, and total *n*-3 PUFA concentrations in erythrocyte lipids, whereas the concentrations of LA, C20:3*n*6, ARA, and total *n*-6 PUFA significantly decreased. Previous studies confirm an increase in EPA, DPA, and DHA in erythrocyte lipids in response to higher intake of *n*-3 long-chain PUFA, at the expense of *n*-6 fatty acids [94,95,96,97].

In the PDI group, the intake of linseed oil (7–9 g/d ALA) resulted in a significant increase in ALA concentration, which was also observed in previous studies [98,99,100]. In addition, there was a significant increase in LA, C18:1c9, and total MUFA concentration, presumably due to higher consumption of nuts, rapeseed, and olive oil.

Despite the reduced intake of SFA (≤7 en%), only a significant reduction in C15:0 was observed in the HTGI group and in C14:0 in both groups. No significant reduction in total SFA content was observed. The PDI group showed an increase in C16:0 and C17:0. Previous studies have demonstrated a positive correlation between LA intake and content in erythrocyte lipids, and a weaker correlation for MUFA and SFA [101,102,103,104]. A possible explanation for this might be that most SFA and MUFA can be synthesized endogenously [101]. However, in the present study, the intake of C18:1c9 and total MUFA was reflected in the erythrocyte lipids. Moreover, the odd-chain fatty acids C15:0 and C17:0 in erythrocyte lipids are considered biomarkers for milk fat intake [105,106,107,108]. In addition, some studies have found a positive correlation between these fatty acids in plasma and fiber intake or the consumption of fruit, vegetables, and seeds. The decrease in C15:0 in the HTGI group could, therefore, indicate the reduction in intake of high-fat dairy products, and the increase in C17:0 in the PDI group could reflect an increased fiber intake [109,110,111,112].

Despite the recommendation to consume an average of 500 mg EPA + DHA daily in the PDI group via high-fat sea fish, the increase in EPA, DPA, and DHA concentrations in the PDI group did not reach statistical significance after 10 weeks. Flock et al. (2013) showed that even a low dose of 300 mg/d EPA + DHA led to a significant increase in EPA and DHA in erythrocyte lipids. However, the intervention was 5 months long [94]. In addition, previous studies have shown that despite the low conversion of ALA to EPA [113], even low ALA intakes of 5 g/d and 3.6 g/d over a shorter period (8 and 6 weeks, respectively) resulted in significantly higher EPA concentrations in erythrocyte lipids [98,99]. A possible reason for this disparity could be that, in contrast to the studies by Kuhnt et al. (2016) and Barceló-Coblijn et al. (2008), the increased LA concentration attenuated the conversion of ALA to EPA, since the same enzymes are used for the conversion of ALA and LA [98,99,114]. In the present study, a significant increase in EPA, DPA, and DHA was observed at follow-up. One possible explanation is that the incorporation of these fatty acids into the erythrocyte membrane occurs mainly during erythropoiesis in the bone marrow. Since erythrocytes have a lifespan of about 120 days [115], which roughly represents the total duration of the study, it accordingly takes a certain time for the circulating erythrocytes to be replaced by newly formed erythrocytes containing EPA, DPA, and DHA, so that a significant increase is measurable.

## 5. Conclusions

The present study shows that combining regular counseling sessions with daily menu plans that provide a diet low in CHO (<50 en%), simple sugars (≤10 en%), SFA (≤7 en%), and high in fiber (>40 g) can lead to a significant reduction in blood lipids, glucose metabolism parameters, FLI, anthropometric parameters, and blood pressure after a 10-week period. The reductions were mostly more pronounced in the intervention groups than in the respective control groups. Except for FLI, there were no differences between the intervention groups. At follow-up, these observed effects were only partially maintained. Our results are consistent with those of previous studies that show that fatty acid concentration in erythrocytes can be considered a biomarker for LA, ALA, EPA, and DHA intake, but not for total SFA.

## 6. Strengths and Limitations

The MoKaRi II study was designed to evaluate the influence of two nutritional concepts on participants with hypertriglyceridemia or prediabetes. Here, it must first be noted that deviating from the defined inclusion criterion of elevated TG concentrations (≥1.7 mmol/L) in the HTGI group, the median was 1.5 (1.1, 2.0) mmol/L. Possible explanations for this deviation from the screening data may be day-to-day fluctuations [2] or observed measurement inaccuracies of the instrument used for the screening (quick test). As a basis for the concepts, menu plans were developed for each day of the interventions, which specified the complete diet of the participants. The menu plans were developed with the help of the nutrition software PRODI, which uses the database of the “Bundeslebensmittelschlüssel” to calculate the nutrient data. Using such menu plans allows a high level of control over energy and nutrient intake of the subjects and can therefore be considered a strength of the study. However, it must always be taken into account that the nutrient profiles of the foods consumed may differ from the underlying database, as seasonal, regional and variety-specific differences as well as preparation can only be considered to a limited extent. The incorporation of the menu plans into the daily routine of the subjects can be demanding, which is why efforts to increase compliance play an important role. Here, the regular counseling sessions on relevant nutrition aspects, on the development of the study parameters and on difficulties in implementing the menu plans and the provision of selected foods and fish oil capsules can be positively highlighted. A final strength of this study was the frequency of study appointments and the analysis of a wide range of parameters, which allowed a comprehensive view of the effects of the interventions over the course of time.

## Figures and Tables

**Table 1:** Characteristics of the study collective in each study arm—Baseline assessment.

Parameters	Hypertriglyceridemia	Prediabetes
Sex	43 w (68.3%), 20 m (31.7%)	47 w (77.0%), 14 m (23.0%)
Age [years]	57.0 (50.5, 63.5)	60.0 (51.0, 66.0)
BMI [kg/m^2^]	30.0 (27.1, 33.6)	29.4 (25.9, 34.3)
Systolic blood pressure [mmHG]	132.0 (123.5, 145.0)	135.0 (125.0, 150.0)
Diastolic blood pressure [mmHG]	88.0 (82.5, 96.0)	87.0 (78.0, 94.0)
Total cholesterol [mmol/L]	6.1 (5.3, 6.6)	5.4 (4.7, 6.1)
LDL cholesterol [mmol/L]	3.8 (3.4, 4.6)	3.4 (2.9, 4.4)
HDL cholesterol [mmol/L]	1.3 (1.2, 1.6)	1.4 (1.3, 1.7)
Non-HDL cholesterol [mmol/L]	4.4 (3.9, 5.3)	3.7 (3.3, 4.7)
Triglycerides [mmol/L]	1.6 (1.2, 2.0)	1.1 (0.8, 1.4)
Blood glucose [mmol/L]	5.8 (5.5, 6.2)	5.8 (5.4, 6.4)
Insulin [mU/L]	11.7 (8.5, 15.9)	11.5 (8.0, 16.2)
C-peptide [ng/mL]	2.5 (2.1, 3.1)	2.5 (1.9, 3.2)
HbA1c [%]	5.8 (5.6, 6.0)	5.8 (5.5, 6.0)

Variables expressed as median (25th, 75th percentile). Abbreviations: BMI, body mass index; C-peptide, connecting peptide; HbA1c, glycated hemoglobin A_1c_; HDL, high-density lipoprotein; LDL, low-density lipoprotein.

**Table 2:** Age and sex of the study collective per group—Baseline assessment.

Parameters	HTGI	HTGC	PDI	PDC
Sex	21 w (70.0%) 9 m (30.0%)	22 w (66.7%) 11 m (33.3%)	23 w (76.7%) 7 m (23.3%)	24 w (77.4%) 7 m (22.6%)
Age [years]	56.5 (48.5, 66.0)	57.0 (51.0, 61.0)	62.5 (51.0, 66.8)	58.0 (51.5, 64.5)

Variable expressed as median (25th, 75th percentile). Abbreviations: HTGC, hypertriglyceridemia control; HTGI, hypertriglyceridemia intervention; PDC, prediabetes control; PDI, prediabetes intervention.

**Table 3:** Cardiovascular and diabetes risk factors and anthropometric measurements at baseline, after the intervention period and at follow-up.

Parameters	Week	HTGI			HTGC			◊	PDI			PDC			◊	●
		*n*	Characteristics *	∆	*n*	Characteristics *	∆		*n*	Characteristics *	∆	*n*	Characteristics *	∆		
Body weight [kg]	0	29	83.4 (76.5, 90.4)	a	27	86.8 (±11.7) 82.6 (78.5, 96.6)	a ^†^	n.s.	30	89.0 (±17.2) 88.2 (75.4, 100.3)	a ^†^	28	78.7 (±18.7)	a ^†^	0.033 ^†^	n.s.
	10		75.9 (70.7, 87.1)	b		86.4 (±11.8) 82.5 (78.8, 96.0)	a ^†^	0.021		83.1 (±15.4) 82.8 (71.0, 90.3)	b ^†^		78.1 (±18.5)	a ^†^	n.s. ^†^	n.s.
	20		76.3 (68.3, 86.9)	b		86.6 (±11.6) 83.3 (78.3, 96.4)	a ^†^	0.014		83.1 (±15.1) 83.0 (72.5, 88.5)	b ^†^		77.7 (±18.3)	a ^†^	n.s. ^†^	n.s.
	%^A→F^	29	−7.3 (±3.9) −7.6 (−9.7, −5.5)		33	−0.5 (±1.4)		<0.001 ^†^	30	−7.5 (−9.3, −4.3)		31	−0.9 (−2.0, 0.6)		<0.001	n.s.
	%^A→G^	30	−7.3 (±5.5) −6.4 (−9.4, −3.2)		33	−0.3 (−1.2, 0.4)		<0.001	30	−6.3 (±4.6)		29	−1.2 (±3.3)		<0.001 ^†^	n.s. ^†^
BMI [kg/m^2^]	0	29	30.9 (±5.0) 30.7 (27.7, 32.9)	a	27	30.7 (±5.1)	a ^†^	n.s. ^†^	30	30.9 (±4.9)	a ^†^	28	28.4 (±6.3)	a ^†^	n.s. ^†^	n.s. ^†^
	10		28.1 (25.6, 29.5)	b		30.5 (±5.0) 29.3 (26.6, 33.8)	a ^†^	n.s.		29.0 (±4.6) 29.0 (25.6, 32.3)	b ^†^		28.2 (±6.2)	a ^†^	n.s. ^†^	n.s.
	20		27.5 (25.2, 29.6)	b		30.6 (±5.0) 29.8 (26.5, 33.8)	a ^†^	n.s.		28.9 (±4.6) 28.8 (25.6, 32.4)	b ^†^		28.0 (±5.9)	a ^†^	n.s. ^†^	n.s.
	%^A→F^	29	−7.4 (±3.9)		33	−0.8 (±1.6)		<0.001 ^†^	30	−6.3 (±3.5)		31	−0.7 (±2.2)		<0.001 ^†^	n.s. ^†^
	%^A→G^	30	−7.4 (±5.6) −6.7 (−10.3, −3.6)		33	−0.3 (−1.2, 0.4)		<0.001	30	−6.4 (±4.5)		29	−1.3 (±3.5)		<0.001 ^†^	n.s. ^†^
Waist circumferences [cm]	0	29	101.6 (±15.7)	a ^†^	27	103.6 (±13.2)	a ^†^	n.s. ^†^	30	103.5 (±11.8)	a ^†^	28	96.1 (±14.6)	a ^†^	0.038 ^†^	n.s. ^†^
	10		95.3 (±15.2)	b ^†^		103.4 (±13.2)	a ^†^	0.039 ^†^		97.3 (±12.3)	b ^†^		95.6 (±14.7)	a ^†^	n.s. ^†^	n.s. ^†^
	20		94.9 (±15.5)	b ^†^		103.0 (±12.1)	a ^†^	0.033 ^†^		97.9 (±10.8)	b ^†^		95.4 (±14.6)	a ^†^	n.s. ^†^	n.s. ^†^
	%^A→F^	29	−6.2 (±3.7)		33	0.2 (±2.8)		<0.001 ^†^	30	−6.1 (±4.3)		31	−0.5 (±2.7)		<0.001 ^†^	n.s. ^†^
	%^A→G^	30	−6.4 (±4.2)		33	−0.4 (±3.7)		<0.001 ^†^	30	−5.3 (±4.2)		29	−1.0 (−2.4, 2.1)		<0.001 ^†^	n.s. ^†^
Systolic blood pressure [mmHG]	0	29	137.4 (±14.2)	a ^†^	27	132.0 (±16.1) 132.0 (119.5, 145.5)	a	n.s. ^†^	30	140.3 (±18.6) 137.0 (127.3, 150.8)	a ^†^	28	130.0 (122.0, 147.3)	a	n.s.	n.s. ^†^
	10		125.4 (±14.0)	b ^†^		131.6 (±18.9) 127.0 (122.0, 138.5)	a	n.s. ^†^		128.6 (±18.0) 129.0 (115.3, 136.8)	b ^†^		126.5 (114.8, 141.0)	b	n.s.	n.s. ^†^
	20		129.8 (±11.5)	b ^†^		131.5 (±18.4) 129.0 (120.0, 139.0)	a	n.s. ^†^		137.2 (±16.8)	a ^†^		135.6 (±17.5) 134.5 (124.0, 144.3)	a	n.s. ^†^	n.s. ^†^
	%^A→F^	29	−8.4 (±9.2)		33	−0.5 (±10.2)		0.002 ^†^	30	−8.1 (±7.9) −8.4 (−12.6, −3.0)		31	−5.5 (−11.2, −1.6)		n.s.	n.s. ^†^
	%^A→G^	30	−4.8 (±7.4)		33	1.0 (±10.4)		0.014 ^†^	30	−1.7 (±8.9) −2.0 (−7.1, 2.7)		29	−1.7 (−6.9, 5.5)		n.s.	n.s. ^†^
Diastolic blood pressure [mmHG]	0	29	87.0 (83.0, 97.0)	a	27	89.0 (82.0, 95.5)	a	n.s.	30	86.9 (±10.7) 88.0 (78.0, 94.8)	a ^†^	28	86.0 (±11.6) 85.0 (77.8, 92.3)	a	n.s. ^†^	n.s.
	10		85.5 (±10.2) 85.0 (78.0, 92.0)	a		89.0 (82.0, 94.5)	a	n.s.		81.0 (±8.3)	b ^†^		80.9 (±9.3) 79.5 (73.8, 89.3)	b	n.s. ^†^	n.s. ^†^
	20		90.1 (±9.8) 91.0 (82.0, 97.0)	a		87.6 (±10.4) 85.0 (79.0, 95.5)	a	n.s. ^†^		85.3 (±9.7)	a ^†^		85.8 (±10.4) 85.5 (78.5, 93.0)	a	n.s. ^†^	n.s. ^†^
	%^A→F^	29	−2.7 (−13.8, 3.6)		33	2.4 (−5.7, 7.5)		n.s.	30	−6.1 (±9.9) −6.3 (−11.1, −1.8)		31	−6.0 (±8.6)		n.s. ^†^	n.s.
	%^A→G^	30	1.6 (−7.7, 11.0)		33	−2.5 (−7.9, 6.6)		n.s.	30	−1.3 (±10.0) −3.6 (−9.0, 5.5)		29	0.4 (±10.0)		n.s. ^†^	n.s.
Total cholesterol [mmol/L]	0	29	6.1 (±1.2)	a ^†^	27	5.9 (±0.8)	a ^†^	n.s. ^†^	30	5.5 (±0.8)	a ^†^	28	5.5 (±1.3) 5.3 (4.5, 6.4)	a	n.s. ^†^	0.025 ^†^
	10		5.0 (±1.0)	b ^†^		5.9 (±0.8)	a ^†^	<0.001 ^†^		4.7 (±0.7) 4.7 (4.2, 5.3)	b ^†^		5.3 (4.5, 6.3)	a	0.038	n.s. ^†^
	20		5.7 (±0.9)	c ^†^		6.0 (±0.9)	a ^†^	n.s. ^†^		5.2 (±1.0) 5.0 (4.6, 5.8)	a ^†^		5.2 (4.7, 6.2)	a	n.s.	n.s. ^†^
	%^A→F^	29	−17.8 (±13.1)		33	0.1 (±9.6)		<0.001 ^†^	30	−13.8 (±9.7) −13.7 (−19.5, −9.5)		31	−0.2 (−5.9, 5.2)		<0.001	n.s. ^†^
	%^A→G^	30	−6.1 (±12.1)		33	1.1 (±8.4)		0.008 ^†^	30	−5.5 (±12.6)		29	1.6 (±7.9)		0.013 ^†^	n.s. ^†^
LDL cholesterol [mmol/L]	0	29	3.9 (±0.9)	a ^†^	27	4.0 (±0.8)	a ^†^	n.s. ^†^	30	3.6 (±0.9) 3.7 (3.0, 4.3)	a ^†^	28	3.2 (2.8, 4.4)	a	n.s.	n.s. ^†^
	10		3.1 (±0.8)	b ^†^		3.9 (±0.7)	a ^†^	<0.001 ^†^		3.1 (±0.7) 3.2 (2.5, 3.6)	b ^†^		3.2 (2.8, 4.1)	a	n.s.	n.s. ^†^
	20		3.6 (±0.8)	c ^†^		4.0 (±0.8)	a ^†^	n.s. ^†^		3.3 (±0.9) 3.3 (2.7, 4.1)	a ^†^		3.4 (2.8, 4.2)	a	n.s.	n.s. ^†^
	%^A→F^	29	−19.8 (±16.6) −19.5 (−27.3, −11.0)		33	−1.3 (−7.0, 4.3)		<0.001	30	−13.0 (±15.3) −14.3 (−22.1, −7.7)		31	0.3 (−6.6, 7.8)		<0.001	n.s. ^†^
	%^A→G^	30	−4.7 (−16.0, 2.7)		33	2.8 (−4.8, 8.8)		0.006	30	−5.7 (±17.3) −6.8 (−12.6, 4.7)		29	4.1 (±11.2)		0.013 ^†^	n.s.
HDL cholesterol [mmol/L]	0	29	1.5 (±0.4) 1.4 (1.2, 1.7)	a	27	1.3 (1.2, 1.6)	a	n.s.	30	1.4 (±0.3) 1.4 (1.2, 1.6)	a ^†^	28	1.6 (1.3, 1.9)	a	0.042	n.s. ^†^
	10		1.3 (±0.3) 1.3 (1.1, 1.5)	b		1.3 (1.1, 1.6)	a	n.s.		1.3 (±0.3) 1.3 (1.1, 1.5)	b ^†^		1.5 (1.3, 1.8)	a	0.012	n.s. ^†^
	20		1.5 (1.2, 1.7)	a		1.4 (1.1, 1.7)	a	n.s.		1.4 (±0.3) 1.4 (1.2, 1.6)	a ^†^		1.6 (±0.5) 1.5 (1.3, 1.8)	a	n.s. ^†^	n.s.
	%^A→F^	29	−9.6 (±12.9) −7.3 (−17.8, −2.3)		33	−3.0 (±8.8)		0.021 ^†^	30	−8.5 (−14.0, 1.2)		31	−3.8 (−8.8, 0.0)		n.s.	n.s.
	%^A→G^	30	0.2 (±9.3) −0.2 (−6.1, 8.3)		33	1.8 (±13.9)		n.s. ^†^	30	1.7 (−6.0, 8.4)		29	−4.3 (−9.4, 1.2)		n.s.	n.s.
Non-HDL cholesterol [mmol/L]	0	29	4.4 (3.7, 5.3)	a	27	4.5 (±0.8) 4.3 (4.0, 5.0)	a ^†^	n.s.	30	4.1 (±0.9) 4.2 (3.5, 4.7)	a ^†^	28	3.8 (±1.2) 3.5 (3.0, 4.9)	a	n.s. ^†^	n.s.
	10		3.7 (±1.0) 3.8 (3.1, 4.2)	b		4.5 (±0.8)	a ^†^	0.002 ^†^		3.4 (±0.7) 3.4 (2.9, 4.0)	b ^†^		3.5 (2.9, 4.6)	a	n.s.	n.s. ^†^
	20		4.2 (±0.9) 4.2 (3.5, 4.7)	a		4.5 (±0.9)	a ^†^	n.s. ^†^		3.8 (±1.0) 3.8 (3.1, 4.6)	b ^†^		3.6 (3.1, 4.4)	a	n.s.	n.s. ^†^
	%^A→F^	29	−19.6 (±16.0) −18.5 (−28.1, −13.8)		33	1.0 (−6.9, 9.1)		<0.001	30	−15.8 (±12.2) −17.6 (−22.6, −7.3)		31	1.7 (−7.3, 9.6)		<0.001	n.s. ^†^
	%^A→G^	30	−7.5 (±15.0) −5.5 (−16.3, −1.4)		33	1.2 (±11.3)		0.011 ^†^	30	−7.9 (−13.1, −0.1)		29	2.3 (−1.5, 11.1)		<0.001	n.s.
Triglycerides [mmol/L]	0	29	1.5 (1.1, 2.0)	a	27	1.7 (1.4, 2.1)	a	n.s.	30	1.3 (1.0, 1.7)	a	28	1.0 (0.8, 1.1)	a	0.003	n.s.
	10		1.1 (0.8, 1.3)	b		1.9 (1.3, 2.5)	a	<0.001		1.1 (0.8, 1.3)	b		1.1 (0.9, 1.4)	a	n.s.	n.s.
	20		1.4 (1.1, 1.9)	a		1.6 (1.2, 2.2)	a	n.s.		1.1 (0.9, 1.7)	a,b		1.1 (0.9, 1.3)	a	n.s.	n.s.
	%^A→F^	29	−18.2 (±44.4) −32.4 (−47.7, 3.6)		33	20.5 (−16.4, 56.3)		0.003	30	−13.0 (±24.6)		31	24.1 (±41.0)		<0.001 ^†^	n.s. ^†^
	%^A→G^	30	−6.6 (−36.8, 41.2)		33	13.1 (−28.0, 44.7)		n.s.	30	−5.9 (±30.2) −10.8 (−24.4, 12.1)		29	16.7 (±28.5)		0.005 ^†^	n.s.
High-sensitivity CRP [mg/L]	0	29	2.2 (1.4, 3.1)	a	27	2.2 (1.6, 3.8)	a	n.s.	30	2.3 (0.9, 3.1)	a	28	1.3 (0.9, 1.8)	a	n.s.	n.s.
	10		1.9 (0.7, 3.2)	b		2.2 (1.8, 4.0)	a	n.s.		1.5 (0.7, 3.5)	a		1.7 (1.0, 2.4)	a	n.s.	n.s.
	20		2.0 (0.6, 4.7)	b		2.4 (1.2, 4.1)	a	n.s.		1.8 (0.4, 4.5)	a		1.2 (0.8, 2.4)	a	n.s.	n.s.
	%^A→F^	29	−20.0 (−55.6, 17.7)		33	20.0 (−5.9, 48.3)		0.006	30	0.0 (−39.9, 10.2)		31	27.3 (−12.4, 50.0)		0.009	n.s.
	%^A→G^	30	−17.0 (−53.1, 0.0)		33	6.7 (−29.4, 33.3)		n.s.	30	−12.8 (−38.9, 43.8)		29	0.0 (−28.6, 28.6)		n.s.	n.s.
Blood glucose [mmol/L]	0	29	5.8 (5.5, 6.1)	a	27	6.0 (5.6, 6.6)	a	n.s.	30	5.8 (5.4, 6.4)	a	27	5.7 (5.4, 6.5)	a	n.s.	n.s.
	10		5.6 (5.3, 5.9)	b		5.8 (5.6, 6.3)	a	0.049		5.6 (5.3, 6.0)	b		5.5 (5.4, 6.0)	a	n.s.	n.s.
	20		5.6 (5.4, 6.2)	a,b		5.8 (5.4, 6.5)	a	n.s.		5.8 (5.5, 6.1)	a,b		5.8 (5.4, 6.1)	a	n.s.	n.s.
	%^A→F^	29	−4.2 (±9.0)		33	−0.2 (±6.7)		0.048 ^†^	30	−5.1 (±9.8)		31	−1.4 (±7.8)		n.s. ^†^	n.s. ^†^
	%^A→G^	30	−0.3 (±8.4)		33	−0.9 (±7.6)		n.s. ^†^	30	−1.9 (±6.6)		29	0.5 (±7.3)		n.s. ^†^	n.s. ^†^
Insulin [mU/L]	0	29	13.3 (±6.3) 12.0 (8.4, 16.3)	a	27	12.0 (±5.9) 10.4 (8.5, 15.5)	a	n.s. ^†^	30	10.2 (8.3, 16.2)	a	28	12.0 (7.1, 16.0)	a	n.s.	n.s.
	10		7.7 (7.3, 10.2)	b		10.7 (8.1, 14.9)	a	n.s.		8.0 (6.1, 11.2)	b		10.6 (8.7, 16.5)	a	0.018	n.s.
	20		10.2 (8.2, 14.3)	a,b		11.2 (6.9, 14.3)	a	n.s.		9.4 (7.4, 10.8)	a,b		10.2 (7.9, 17.3)	a	n.s.	n.s.
	%^A→F^	29	−26.8 (−41.2, −11.9)		33	1.0 (−20.2, 19.2)		<0.001	30	−17.7 (−46.9, −5.5)		31	11.3 (−7.0, 39.9)		<0.001	n.s.
	%^A→G^	30	−21.6 (−27.7, 15.6)		33	−9.3 (−24.4, 18.1)		n.s.	30	−8.0 (−29.9, 3.3)		29	8.0 (−9.1, 23.9)		0.017	n.s.
C-peptide [ng/mL]	0	29	2.7 (±0.8) 2.6 (2.1, 3.0)	a	27	2.7 (±0.9)	a ^†^	n.s. ^†^	30	2.6 (±0.8) 2.5 (1.9, 3.2)	a	28	2.5 (±1.0) 2.5 (1.7, 3.2)	a	n.s. ^†^	n.s. ^†^
	10		2.0 (1.8, 2.3)	b		2.6 (±0.7) 2.7 (2.2, 3.1)	a ^†^	0.025		2.1 (1.8, 2.6)	b		2.3 (1.9, 3.1)	a	n.s.	n.s.
	20		2.4 (±0.9) 2.2 (1.9, 2.6)	b		2.8 (±0.9)	a ^†^	n.s. ^†^		2.2 (±0.6) 2.2 (1.9, 2.5)	b		2.2 (1.7, 3.1)	a	n.s.	n.s. ^†^
	%^A→F^	29	−14.8 (−25.0, −9.1)		33	0.0 (−9.7, 11.8)		<0.001	30	−15.1 (−25.8, −4.7)		31	3.2 (−6.8, 20.1)		<0.001	n.s.
	%^A→G^	30	−11.0 (±20.4)		33	3.1 (±21.6)		0.01 ^†^	30	−11.9 (±14.6)		29	−0.6 (±19.4)		0.015 ^†^	n.s. ^†^
HOMA-IR	0	29	3.3 (2.5, 4.1)	a	27	2.9 (2.0, 4.6)	a	n.s.	30	2.8 (2.2, 4.4)	a	27	3.4 (1.8, 4.6)	a	n.s.	n.s.
	10		1.9 (1.7, 2.7)	b		3.0 (2.0, 3.8)	a	n.s.		2.1 (1.5, 3.1)	b		2.6 (2.1, 4.2)	a	0.022	n.s.
	20		2.5 (1.9, 3.8)	a,b		2.9 (1.9, 3.7)	a	n.s.		2.4 (2.1, 3.1)	a,b		2.7 (1.9, 4.3)	a	n.s.	n.s.
	%^A→F^	29	−31.8 (−46.1, −10.1)		33	2.9 (−25.4, 23.2)		<0.001	30	−26.4 (−48.3, −6.6)		31	11.2 (−10.0, 45.3)		<0.001	n.s.
	%^A→G^	30	−21.0 (−35.6, 11.2)		33	−9.3 (−23.8, 16.4)		n.s.	30	−12.2 (−33.4, 9.7)		29	7.5 (−15.8, 30.7)		0.029	n.s.
HbA1c [%]	0	29	5.9 (5.7, 6.1)	a	27	5.8 (5.7, 6.0)	a	n.s.	30	5.8 (±0.4) 5.8 (5.6, 6.1)	a ^†^	28	5.7 (5.5, 5.9)	a	n.s.	n.s.
	10		5.7 (±0.3) 5.8 (5.6, 5.8)	b		5.7 (±0.3) 5.8 (5.6, 5.9)	b	n.s. ^†^		5.6 (±0.3) 5.6 (5.4, 5.7)	b ^†^		5.7 (5.4, 5.8)	a	n.s.	n.s. ^†^
	20		5.7 (5.7, 5.9)	a		5.8 (5.7, 6.0)	a	n.s.		5.7 (±0.3) 5.7 (5.4, 5.9)	c ^†^		5.7 (±0.3) 5.7 (5.5, 5.9)	a	n.s. ^†^	n.s.
	%^A→F^	29	−3.4 (−5.1, −1.8)		33	0.0 (−3.3, 1.7)		0.003	30	−5.0 (−5.3, −2.3)		31	−1.7 (−3.3, 0.9)		<0.001	n.s.
	%^A→G^	30	−1.7 (−3.3, 0.0)		33	0.0 (0.0, 1.8)		0.005	30	−1.9 (−4.6, −1.6)		29	0.0 (−1.8, 1.8)		0.01	n.s.
TyG index [mg/dL]	0	29	8.9 (±0.5) 8.8 (8.5, 9.2)	a	27	9.0 (±0.5)	a ^†^	n.s. ^†^	30	8.8 (±0.5) 8.8 (8.4, 9.0)	a	28	8.4 (±0.4)	a ^†^	0.002 ^†^	n.s. ^†^
	10		8.5 (8.1, 8.7)	b		9.0 (±0.5) 9.1 (8.6, 9.3)	a ^†^	<0.001		8.5 (8.3, 8.7)	b		8.5 (±0.5) 8.5 (8.2, 8.8)	a ^†^	n.s.	n.s.
	20		8.7 (8.4, 9.1)	a		8.9 (±0.5) 8.9 (8.6, 9.3)	a ^†^	n.s.		8.6 (±0.6) 8.5 (8.3, 8.9)	a,b		8.5 (±0.4)	a ^†^	n.s. ^†^	n.s.
	%^A→F^	29	−4.4 (±6.3)		33	1.2 (±6.1)		0.001 ^†^	30	−2.6 (±3.3)		31	1.8 (±4.1)		<0.001 ^†^	n.s. ^†^
	%^A→G^	30	−0.5 (±7.2)		33	−0.1 (±6.6)		n.s. ^†^	30	−1.5 (±3.8)		29	1.6 (±3.3)		0.002 ^†^	n.s. ^†^
FLI	0	29	70.0 (45.8, 89.6)	a	33	70.3 (53.1, 88.1)	a	n.s.	30	71.6 (47.8, 91.4)	a	29	48.1 (22.7, 78.9)	a	0.022	n.s.
	10		38.5 (16.3, 63.0)	b		69.2 (54.2, 90.1)	a	0.002		53.4 (25.8, 73.1)	b		50.6 (22.4, 79.4)	a	n.s.	n.s.
	20		50.9 (20.0, 78.9)	c		65.0 (48.5, 90.6)	a	0.029		58.4 (26.4, 81.2)	b		44.3 (27.1, 77.4)	a	n.s.	n.s.
	%^A→F^	29	−38.3 (−64.8, −20.9)		33	0.7 (−2.1, 14.5)		<0.001	30	−25.3 (−44.6, −10.8)		31	2.6 (−6.4, 24.5)		<0.001	0.035
	%^A→G^	30	−15.9 (−55.2, −2.1)		33	1.9 (−5.4, 9.3)		<0.001	30	−12.8 (−30.5, −4.9)		29	4.9 (−2.3, 24.9)		<0.001	n.s.

* Variables expressed as mean (±SD) and/or as median (25th, 75th percentile) depending on the statistical test that was performed; ∆ Differences within groups comparing points in time, points in time without a common letter are significantly different, *p* < 0.05; **◊** Differences between each intervention group and their corresponding control group; **●** Differences between both intervention groups; %^A→F^, percentage change from baseline to week 10; %^A→G^, percentage change from baseline to follow-up; ^†^ Calculated with parametric test. Abbreviations: BMI, body mass index; CRP, c-reactive protein; C-peptide, connecting peptide; FLI, fatty liver index; HbA1c, glycated hemoglobin A_1c_; HDL, high-density lipoprotein; HOMA-IR, Homeostatic Model Assessment for Insulin Resistance; HTGC, hypertriglyceridemia control; HTGI, hypertriglyceridemia intervention; LDL, low-density lipoprotein; PDC, prediabetes control; PDI, prediabetes intervention; TyG, triglyceride glucose.

**Table 4:** Bioelectrical impedance analysis at baseline, after the intervention period and at follow-up.

Parameters	Week	HTGI			HTGC			◊	PDI			PDC			◊
		*n*	Characteristics *	∆	*n*	Characteristics *	∆		*n*	Characteristics *	∆	*n*	Characteristics *	∆	
Body fat [kg]	0	29	34.6 (27.6, 38.2)	a	23	33.9 (±11.9) 33.7 (24.6, 40.2)	a ^†^	n.s.	30	33.8 (±12.4)	a ^†^	26	27.8 (±13.0)	a ^†^	n.s. ^†^
	10		29.4 (23.9, 31.1)	b		32.7 (±11.5) 32.0 (24.7, 37.5)	a ^†^	n.s.		29.0 (±11.4)	b ^†^		27.3 (±12.6)	a ^†^	n.s. ^†^
	20		28.2 (24.5, 35.4)	b		34.1 (±12.2) 33.3 (25.5, 40.0)	a ^†^	n.s.		29.0 (±11.2)	b ^†^		26.8 (±12.3)	a ^†^	n.s. ^†^
	%^A→F^	20	−14.7 (−19.7, −12.1)		30	−2.3 (−5.3, 0.5)		<0.001	30	−15.2 (±11.1)		30	−1.7 (±6.0)		<0.001 ^†^
	%^A→G^	30	−12.9 (±11.0)		31	0.2 (±4.8)		<0.001 ^†^	30	−14.6 (−22.0, −6.6)		28	−4.3 (−7.2, 1.8)		<0.001
Body fat [%]	0	29	40.5 (±8.1)	a ^†^	23	38.7 (±9.8)	a ^†^	n.s. ^†^	30	39.4 (32.0, 43.9)	a	26	33.9 (±10.1) 35.2 (28.2, 39.7)	a ^†^	n.s.
	10		37.3 (±8.4)	b ^†^		37.6 (±9.4)	a ^†^	n.s. ^†^		36.5 (27.4, 42.5)	b		33.6 (±9.9) 34.9 (27.2, 39.1)	a ^†^	n.s.
	20		37.8 (±7.7)	b ^†^		38.8 (±10.1)	a ^†^	n.s. ^†^		37.3 (26.7, 41.9)	b		33.3 (±9.9) 33.3 (27.1, 38.8)	a ^†^	n.s.
	%^A→F^	20	−7.6 (−12.2, −3.8)		30	−1.7 (−4.4, 0.4)		<0.001	30	−8.3 (−13.3, −4.6)		30	−1.2 (−2.8, 0.4)		<0.001
	%^A→G^	30	−6.4 (±7.0)		31	0.3 (±3.9)		<0.001 ^†^	30	−8.4 (−11.3, −3.8)		28	−2.3 (−3.9, 0.9)		<0.001
Body water [l]	0	29	35.7 (32.3, 42.4)	a	23	37.1 (34.5, 43.5)	a	n.s.	30	37.7 (35.4, 44.1)	a	26	35.4 (31.1, 40.7)	a	n.s.
	10		34.3 (31.6, 42.8)	b		39.1 (34.8, 43.9)	a	n.s.		37.4 (34.4, 43.7)	b		34.9 (31.7, 40.3)	a	n.s.
	20		34.4 (31.2, 41.0)	b		38.2 (34.9, 44.0)	a	n.s.		37.0 (34.6, 43.4)	b		34.8 (31.6, 40.00)	a	n.s.
	%^A→F^	20	−2.8 (−3.9, −1.3)		30	0.9 (0.1, 1.8)		<0.001	30	−2.1 (−4.4, −0.7)		30	−0.7 (−1.9, 0.9)		0.030
	%^A→G^	30	−3.3 (−5.4, −1.1)		31	−0.3 (−1.7, 1.2)		<0.001	30	−3.1 (±3.2)		28	−0.8 (±3.5)		0.012 ^†^
Lean body mass [kg]	0	29	47.5 (42.4, 57.6)	a	23	48.9 (46.1, 58.7)	a	n.s.	30	51.5 (48.3, 60.2)	a	26	48.4 (42.6, 55.6)	a	n.s.
	10		46.1 (42.3, 58.4)	b		51.3 (46.5, 59.5)	a	n.s.		51.0 (46.9, 59.7)	b		47.7 (43.3, 55.2)	a	n.s.
	20		45.9 (41.9, 56.1)	b		50.2 (46.1, 58.5)	a	n.s.		50.5 (47.3, 59.3)	b		47.4 (43.1, 54.7)	a	n.s.
	%^A→F^	20	−2.1 (−4.1, −1.0)		30	0.7 (0.3, 1.9)		<0.001	30	−2.1 (−4.2, −0.8)		30	−0.8 (−2.0, 0.7)		0.020
	%^A→G^	30	−2.4 (−5.2, −0.8)		31	−0.4 (−1.5, 0.5)		0.001	30	−3.1 (±3.3)		28	−0.8 (±3.5)		0.013 ^†^

* Variables expressed as mean (±SD) and/or as median (25th, 75th percentile) depending on the statistical test that was performed; **∆** Differences within groups comparing points in time, points in time without a common letter are significantly different, *p* < 0.05; **◊** Differences between each intervention group and their corresponding control group; %^A→F^, percentage change from baseline to week 10; %^A→G^, percentage change from baseline to follow-up; ^†^ Calculated with parametric test. Abbreviations: HTGC, hypertriglyceridemia control; HTGI, hypertriglyceridemia intervention; PDC, prediabetes control; PDI, prediabetes intervention.

**Table 5:** Analyses for visceral adipose tissue in the HTGI and HTGC group at baseline, after the intervention period and at follow-up.

Parameters	Week	HTGI			HTGC			◊
		n	Characteristics *	∆	n	Characteristics *	∆	
Visceral adipose tissue [l]	0	23	2.6 (1.9, 3.6)	a	19	2.4 (2.0, 4.4)	a	n.s.
	10		1.9 (1.4, 3.0)	b		2.5 (1.9, 4.6)	a	0.037
	20		2.0 (1.2, 2.8)	b		2.6 (1.8, 4.2)	a	0.032
	%^A→F^	29	−22.8 (±14.4)		29	0.7 (±13.0)		<0.001 ^†^
	%^A→G^	30	−24.0 (±15.8)		30	0.1 (±17.5)		<0.001 ^†^

* Variables expressed as mean (±SD) and/or as median (25th, 75th percentile) depending on the statistical test that was performed; ∆ Differences within groups comparing points in time, points in time without a common letter are significantly different, *p* < 0.05; ◊ Differences between groups; %^A→F^, percentage change from baseline to week 10; %^A→G^, percentage change from baseline to follow-up; ^†^ Calculated with parametric test. Abbreviations: HTGC, hypertriglyceridemia control; HTGI, hypertriglyceridemia intervention.

**Table 6:** Body weight (kg), LDL cholesterol (mmol/L), TG (mmol/L), and HOMA-IR at baseline and the minimum values within the intervention period.

Parameters	Week	HTGI (n = 29)		PDI (n = 30)		●
		Characteristics *	∆	Characteristics *	∆	
Body weight [kg]	0	83.4 (76.5, 90.4)		89.0 (±17.2) 88.2 (75.4, 100.3)		n.s.
	Min Int	75.9 (70.7, 86.4)	<0.001	82.9 (±15.4) 82.8 (70.6, 90.3)	<0.001 ^†^	n.s.
	Min Cfb	−6.6 (±3.4)		−6.1 (±3.4)		n.s. ^†^
	Min Cfb [%]	−7.6 (±3.6)		−6.7 (±3.2)		n.s. ^†^
LDL cholesterol [mmol/L]	0	3.9 (±0.9)		3.6 (±0.9)		n.s. ^†^
	Min Int	2.7 (±0.6)	<0.001 ^†^	2.8 (±0.7)	<0.001 ^†^	n.s. ^†^
	Min Cfb	−1.0 (−1.5, −0.8)		−0.7 (−1.0, −0.6)		0.004
	Min Cfb [%]	−27.2 (−35.5, −23.0)		−20.5 (−27.6, −15.4)		0.004
Triglycerides [mmol/L]	0	1.5 (1.1, 2.0)		1.3 (1.0, 1.7)		n.s.
	Min Int	0.9 (0.7, 1.1)	<0.001	0.9 (0.8, 1.1)	<0.001	n.s.
	Min Cfb	−0.6 (−1.1, −0.2)		−0.3 (−0.7, −0.1)		n.s.
	Min Cfb [%]	−30.3 (±39.9)		−28.3 (±20.5)		n.s. ^†^
HOMA-IR	0	3.3 (2.5, 4.1)		2.8 (2.2, 4.4)		n.s.
	Min Int	1.6 (1.2, 2.1)	<0.001	1.9 (1.3, 2.3)	<0.001	n.s.
	Min Cfb	−1.3 (−2.1, −0.8)		−1.1 (−1.8, −0.5)		n.s.
	Min Cfb [%]	−47.4 (±18.5)		−38.7 (±22.9)		n.s. ^†^

* Variables expressed as mean (±SD) and/or as median (25th, 75th percentile) depending on the statistical test that was performed; ∆ Differences within groups; ● Differences between both intervention groups; Min Int, minimum value in the intervention period; Min Cfb, absolute change between baseline and minimum value; Min Cfb [%], percentage change between baseline and minimum value; ^†^ Calculated with parametric test. Abbreviations: HOMA-IR, Homeostatic Model Assessment for Insulin Resistance; HTGI, hypertriglyceridemia intervention; LDL, low-density lipoprotein; PDI, prediabetes intervention.

**Table 7:** Distribution of minimum values for body weight (kg), LDL cholesterol (mmol/L), TG (mmol/L), and HOMA-IR over the intervention period.

Parameters	Week	HTGI		PDI	
		n	%	n	%
Body weight [kg]	2	0	0.0	0	0.0
	4	1	3.4	1	3.3
	6	2	6.9	1	3.3
	8	4	13.8	7	23.3
	10	22	75.9	21	70.0
LDL cholesterol [mmol/L]	2	9	31.0	10	33.3
	4	3	10.3	8	26.7
	6	5	17.2	3	10.0
	8	4	13.8	3	10.0
	10	8	27.6	6	20.0
Triglycerides [mmol/L]	2	3	10.3	12	40.0
	4	9	31.0	3	10.0
	6	4	13.8	6	20.0
	8	4	13.8	3	10.0
	10	9	31.0	6	20.0
HOMA-IR	2	2	6.9	5	16.7
	4	8	27.6	5	16.7
	6	2	6.9	8	26.7
	8	6	20.7	5	16.7
	10	11	37.9	7	23.3

Abbreviations: HOMA-IR, Homeostatic Model Assessment for Insulin Resistance; HTGC, hypertriglyceridemia control; HTGI, hypertriglyceridemia intervention; LDL, low-density lipoprotein; PDC, prediabetes control; PDI, prediabetes intervention.

**Table 8:** Erythrocyte fatty acids at baseline, after the intervention period and at follow-up.

Parameters [% FAME]	Week	HTGI			HTGC			◊	PDI			PDC			◊	●
		n	Characteristics *	∆	n	Characteristics *	∆		n	Characteristics *	∆	n	Characteristics *	∆		
C14:0	0	26	0.3 (±0.1) 0.3 (0.3, 0.4)	a	28	0.3 (±0.1)	a ^†^	n.s. ^†^	29	0.3 (±0.1) 0.3 (0.3, 0.4)	a	27	0.3 (±0.1)	a ^†^	n.s. ^†^	n.s. ^†^
	10		0.2 (0.2, 0.2)	b		0.3 (±0.1) 0.3 (0.3, 0.4)	b ^†^	<0.001		0.2 (0.2, 0.3)	b		0.31 (±0.1) 0.3 (0.3, 0.4)	a ^†^	<0.001	n.s.
	20		0.3 (±0.1) 0.3 (0.2, 0.3)	a		0.3 (±0.1)	b ^†^	n.s. ^†^		0.3 (±0.1) 0.3 (0.2, 0.3)	a		0.3 (±0.1)	a ^†^	n.s. ^†^	n.s. ^†^
	%^A→F^	27	−34.6 (±19.9) −40.1 (−49.0, −18.0)		28	9.7 (±22.6)		<0.001 ^†^	29	−32.0 (−42.8, −11.9)		29	24.0 (−5.9, 38.0)		<0.001	n.s.
	%^A→G^	28	1.5 (−17.5, 14.4)		30	13.4 (−0.6, 32.3)		0.032	29	−15.5 (−33.2, 5.1)		28	11.1 (−13.0, 32.6)		0.013	n.s.
C15:0	0	28	0.2 (±0.0) 0.2 (0.1, 0.2)	a ^†^	30	0.2 (0.1, 0.2)	a	n.s.	30	0.2 (0.2, 0.2)	a,b	27	0.2 (±0.0) 0.2 (0.1, 0.2)	a ^†^	n.s.	n.s.
	10		0.2 (±0.0)	b ^†^		0.2 (±0.0) 0.2 (0.1, 0.2)	a	n.s. ^†^		0.2 (±0.0) 0.2 (0.2, 0.2)	a		0.2 (±0.0)	a ^†^	n.s. ^†^	0.015 ^†^
	20		0.2 (±0.0)	a ^†^		0.2 (±0.0) 0.2 (0.1, 0.2)	a	n.s. ^†^		0.2 (±0.0) 0.2 (0.2, 0.2)	b		0.2 (±0.0)	a ^†^	n.s. ^†^	n.s. ^†^
	%^A→F^	28	−13.0 (−26.3, −4.2)		30	−2.4 (−10.8, 9.0)		0.043	30	2.8 (−7.5, 11.6)		29	7.4 (−4.8, 21.9)		n.s.	0.003
	%^A→G^	30	−4.5 (±17.4) −0.9 (−13.4, 4.7)		32	1.4 (±22.8)		n.s. ^†^	30	−3.8 (−12.1, 6.2)		28	1.4 (−7.4, 9.7)		n.s.	n.s.
C16:0	0	28	21.3 (20.8, 22.7)	a	30	21.0 (19.5, 21.6)	a	n.s.	30	20.2 (19.3, 21.5)	a	27	20.3 (19.2, 22.3)	a	n.s.	0.042
	10		21.4 (±2.6) 21.4 (19.5, 23.3)	a		20.8 (±2.0) 20.9 (19.2, 21.9)	a	n.s. ^†^		21.6 (20.8, 22.2)	b		21.5 (20.6, 22.5)	a	n.s.	n.s.
	20		20.6 (±1.4) 20.6 (19.8, 21.4)	a		20.7 (19.3, 21.6)	a	n.s.		19.7 (±1.1) 19.8 (19.0, 20.5)	c		19.4 (±1.0) 19.6 (18.7, 20.0)	b	n.s. ^†^	0.005 ^†^
	%^A→F^	28	−0.9 (±18.2) −2.0 (−11.7, 12.2)		30	1.1 (±11.5)		n.s. ^†^	30	3.9 (−2.3, 14.8)		29	5.0 (−3.9, 25.7)		n.s.	n.s.
	%^A→G^	30	−3.9 (−15.3, 3.3)		32	−2.0 (−7.0, 7.3)		n.s.	30	−6.6 (−8.8, 1.0)		28	−3.4 (−11.6, −1.3)		n.s.	n.s.
C17:0	0	28	0.3 (±0.1)	a ^†^	30	0.3 (±0.0)	a ^†^	0.049 ^†^	30	0.3 (±0.0) 0.3 (0.2, 0.3)	a ^†^	27	0.3 (0.2, 0.3)	a	n.s.	n.s. ^†^
	10		0.3 (±0.0)	a ^†^		0.3 (±0.0)	a ^†^	0.004 ^†^		0.3 (±0.0) 0.3 (0.3, 0.3)	b ^†^		0.3 (0.3, 0.3)	b	n.s.	n.s. ^†^
	20		0.3 (±0.0)	a ^†^		0.3 (±0.1)	a ^†^	n.s. ^†^		0.3 (±0.0)	c ^†^		0.3 (±0.0) 0.3 (0.3, 0.3)	a,b	n.s. ^†^	n.s. ^†^
	%^A→F^	28	8.0 (±21.0)		30	2.9 (±13.2)		n.s. ^†^	30	18.0 (±21.2) 17.3 (10.2, 23.5)		29	6.3 (2.7, 22.3)		n.s.	n.s. ^†^
	%^A→G^	30	4.8 (±17.2) 6.3 (−6.1, 16.6)		32	5.7 (−0.1, 14.2)		n.s.	30	9.6 (±14.0) 11.2 (2.4, 17.6)		28	5.2 (−1.8, 10.3)		0.032	n.s. ^†^
C18:0	0	28	11.2 (10.5, 13.4)	a	30	11.5 (10.4, 12.6)	a	n.s.	30	11.2 (10.5, 12.3)	a,b	27	12.4 (10.3, 13.3)	a	n.s.	n.s.
	10		11.9 (10.8, 12.6)	a		12.4 (11.6, 13.2)	b	n.s.		10.4 (9.7, 11.6)	a		11.0 (10.4, 12.4)	a	0.018	0.002
	20		11.0 (10.1, 12.1)	a		11.4 (10.6, 13.7)	a,b	n.s.		11.7 (±1.5) 11.5 (10.6, 12.8)	b		12.0 (±1.3) 11.8 (10.9, 12.9)	a	n.s. ^†^	n.s.
	%^A→F^	28	7.0 (±28.8)		30	9.0 (±21.1)		n.s. ^†^	30	−10.0 (±23.2) −8.6 (−20.2, 2.6)		29	−4.1 (−16.0, 18.2)		n.s.	0.016 ^†^
	%^A→G^	30	0.7 (±22.7) −1.3 (−16.7, 15.3)		32	6.0 (±25.9)		n.s. ^†^	30	4.6 (−14.5, 15.7)		28	−0.7 (−12.9, 15.4)		n.s.	n.s.
C18:1c9	0	27	14.5 (13.8, 15.3)	a	30	14.4 (13.8, 15.1)	a	n.s.	30	14.1 (12.9, 15.0)	a	27	13.6 (13.0, 14.5)	a	n.s.	n.s.
	10		14.3 (13.8, 15.2)	a		14.7 (13.6, 15.6)	a	n.s.		15.2 (14.6, 15.7)	b		14.7 (13.7, 15.4)	b	n.s.	0.015
	20		14.8 (±1.5) 15.0 (14.0, 16.0)	a		14.9 (13.2, 15.3)	a	n.s.		14.9 (±1.1) 14.8 (14.4, 15.4)	a,b		14.6 (±1.0) 14.7 (14.1, 15.1)	b	n.s. ^†^	n.s. ^†^
	%^A→F^	27	0.1 (−10.5, 5.5)		30	2.3 (−6.5, 7.2)		n.s.	30	8.6 (0.1, 15.1)		29	7.6 (2.7, 17.3)		n.s.	0.006
	%^A→G^	29	2.6 (−2.5, 10.6)		32	−0.7 (−6.6, 6.7)		n.s.	30	2.2 (−0.2, 9.2)		28	4.0 (0.1, 16.9)		n.s.	n.s.
C18:2c9,c12 (LA)	0	28	10.1 (±1.5)	a ^†^	30	9.6 (±1.7) 10.0 (8.5, 10.4)	a	n.s. ^†^	30	9.8 (±2.3)	a ^†^	27	10.4 (±1.7) 10.2 (9.4, 11.5)	a	n.s. ^†^	n.s. ^†^
	10		8.4 (±1.7) 8.4 (7.2, 9.6)	b ^†^		10.2 (8.8, 11.2)	a	0.003		11.2 (±1.8) 11.1 (9.8, 12.0)	b ^†^		11.0 (10.2, 12.2)	a	n.s.	<0.001 ^†^
	20		10.4 (±1.5)	a ^†^		10.0 (±1.4) 10.1 (9.1, 10.9)	a	n.s. ^†^		10.8 (±1.3)	a,b ^†^		10.8 (±1.4) 10.6 (9.9, 11.4)	a	n.s. ^†^	n.s. ^†^
	%^A→F^	28	−14.8 (±23.9) −14.5 (−32.2, −2.3)		30	2.8 (±19.5)		0.003 ^†^	30	10.8 (−2.0, 34.6)		29	5.6 (−4.0, 20.3)		n.s.	<0.001
	%^A→G^	30	4.7 (−8.0, 13.2)		32	1.9 (−8.1, 15.4)		n.s.	30	6.5 (−0.5, 22.5)		28	3.2 (−4.7, 16.1)		n.s.	n.s.
aC18:3c9,c12,c15 (ALA)	0	28	0.2 (0.1, 0.2)	a	30	0.2 (0.2, 0.2)	a	n.s.	30	0.2 (0.1, 0.2)	a	27	0.2 (0.1, 0.2)	a	n.s.	n.s.
	10		0.2 (0.1, 0.2)	a		0.2 (0.2, 0.2)	a	n.s.		0.3 (0.2, 0.3)	b		0.2 (0.1, 0.2)	a	<0.001	<0.001
	20		0.2 (0.1, 0.2)	a		0.2 (0.1, 0.2)	a	n.s.		0.2 (0.2, 0.3)	c		0.2 (0.1, 0.2)	a	0.008	0.010
	%^A→F^	28	−1.1 (±40.0) −6.1 (−28.0, 17.3)		30	0.4 (−19.8, 18.5)		n.s.	30	66.7 (±67.2)		29	11.0 (±32.5)		<0.001 ^†^	<0.001 ^†^
	%^A→G^	30	−0.1 (±41.8) 1.1 (−33.7, 19.7)		32	−7.4 (±26.1)		n.s. ^†^	30	27.0 (−14.1, 50.2)		28	1.2 (−22.2, 29.4)		n.s.	<0.001
CLA-c9,t11/t8,c10	0	28	0.1 (0.1, 0.2)	a	29	0.1 (0.1, 0.2)	a	n.s.	30	0.1 (0.1, 0.2)	a	27	0.1 (0.1, 0.1)	a	n.s.	n.s.
	10		0.1 (0.1, 0.1)	a,b		0.1 (0.1, 0.1)	a	n.s.		0.1 (0.1, 0.1)	b		0.1 (0.1, 0.1)	a	n.s.	n.s.
	20		0.1 (±0.0) 0.1 (0.1, 0.1)	b		0.1 (±0.0) 0.1 (0.1, 0.1)	a	0.032 ^†^		0.1 (±0.0) 0.1 (0.1, 0.1)	a,b		0.1 (±0.0) 0.1 (0.1, 0.1)	a	n.s. ^†^	n.s. ^†^
	%^A→F^	28	−16.0 (−52.5, 37.2)		29	−10.7 (−39.5, 17.1)		n.s.	30	−22.1 (−46.9, 10.2)		29	−14.4 (−29.1, 1.0)		n.s.	n.s.
	%^A→G^	30	−25.5 (−42.3, −0.4)		31	−22.0 (−43.6, 20.2)		n.s.	30	−11.7 (−33.5, 10.0)		28	−1.9 (−20.7, 8.7)		n.s.	n.s.
DGLA	0	28	1.7 (1.6, 1.9)	a	30	1.7 (1.4, 2.0)	a	n.s.	30	1.7 (±0.4) 1.7 (1.4, 1.9)	a	27	1.6 (±0.3) 1.6 (1.4, 1.8)	a	n.s. ^†^	n.s.
	10		1.2 (±0.4) 1.2 (1.0, 1.5)	b		1.6 (±0.3) 1.6 (1.4, 1.8)	a	<0.001 ^†^		1.5 (±0.4) 1.5 (1.2, 1.6)	b		1.5 (±0.3) 1.5 (1.3, 1.7)	b	n.s. ^†^	0.019 ^†^
	20		1.5 (±0.3) 1.5 (1.3, 1.7)	c		1.6 (±0.3) 1.6 (1.4, 1.8)	a	n.s. ^†^		1.6 (1.4, 1.9)	a		1.5 (1.4, 1.8)	a	n.s.	n.s.
	%^A→F^	28	−30.0 (−49.0, −14.9)		30	−8.4 (−14.0, 5.1)		<0.001	30	−11.2 (−24.9, 1.7)		29	−8.1 (−17.2, 2.7)		n.s.	0.001
	%^A→G^	30	−14.3 (±18.7)		32	−2.3 (±22.6)		0.027 ^†^	30	2.9 (±20.1) −1.3 (−10.1, 14.7)		28	−0.4 (−6.2, 14.5)		n.s.	0.001 ^†^
C20:4c5,c8,c11,c14 (ARA)	0	28	13.0 (±1.5) 13.2 (12.2, 13.9)	a ^†^	30	13.3 (±1.7) 13.5 (11.8, 14.3)	a	n.s. ^†^	30	13.4 (11.7, 14.3)	a,b	27	12.9 (11.5, 14.5)	a,b	n.s.	n.s.
	10		11.1 (±1.6) 11.2 (9.6, 12.3)	b ^†^		12.4 (±1.5) 12.3 (11.8, 13.6)	a	0.002 ^†^		11.9 (11.1, 12.8)	a		12.3 (11.5, 13.2)	a	n.s.	n.s.
	20		11.9 (±1.3) 12.0 (11.2, 12.7)	c ^†^		12.9 (11.5, 13.8)	a	0.034		13.1 (±1.1) 13.4 (12.1, 13.9)	b		13.3 (±1.6) 13.3 (12.5, 13.8)	b	n.s. ^†^	<0.001 ^†^
	%^A→F^	28	−12.4 (±19.7) −16.4 (−22.0, −3.8)		30	−5.8 (±13.8)		n.s. ^†^	30	−7.7 (−15.2, 0.4)		29	−5.0 (−18.1, 4.2)		n.s.	n.s.
	%^A→G^	30	−5.9 (±13.8) −7.4 (−13.9, 5.5)		32	−4.8 (±16.2)		n.s. ^†^	30	1.6 (−9.4, 10.9)		28	0.8 (−7.1, 10.0)		n.s.	0.043
C20:4*n*3 (ETA)	0	26	0.1 (0.1, 0.2)	a	28	0.1 (0.1, 0.2)	a	n.s.	30	0.1 (0.1, 0.2)	a	27	0.1 (0.1, 0.2)	a	n.s.	n.s.
	10		0.1 (0.1, 0.2)	a		0.1 (0.1, 0.2)	a	n.s.		0.1 (0.1, 0.2)	a		0.1 (0.1, 0.1)	a	n.s.	n.s.
	20		0.1 (0.1, 0.1)	a		0.1 (0.1, 0.2)	a	0.002		0.1 (0.1, 0.2)	a		0.1 (0.1, 0.1)	a	n.s.	n.s.
	%^A→F^	26	−5.1 (−55.1, 75.2)		28	−36.2 (−56.8, 70.8)		n.s.	30	−8.9 (−37.9, 41.7)		29	−16.7 (−54.7, 19.6)		n.s.	n.s.
	%^A→G^	29	−36.1 (−62.1, 27.0)		31	3.1 (−40.6, 70.3)		n.s.	30	−23.7 (−46.4, 23.6)		28	−23.2 (−40.4, 60.7)		n.s.	n.s.
C20:5*n*3 (EPA)	0	28	0.8 (0.7, 1.1)	a	30	0.9 (0.7, 1.1)	a	n.s.	30	0.7 (0.7, 0.9)	a	27	0.8 (0.5, 1.0)	a	n.s.	n.s.
	10		2.7 (±0.8) 2.8 (2.2, 3.1)	b		0.9 (0.7, 1.0)	a	<0.001		0.8 (±0.2) 0.8 (0.7, 1.0)	a,b		0.7 (0.6, 0.9)	a	n.s.	<0.001 ^†^
	20		1.6 (1.1, 1.9)	c		1.0 (0.8, 1.2)	a	<0.001		0.9 (0.7, 1.1)	b		0.8 (0.7, 1.0)	a	n.s.	<0.001
	%^A→F^	28	211.4 (±122.2) 219.1 (119.7, 270.0)		30	−3.4 (−21.9, 18.0)		<0.001	30	3.7 (±33.4) 3.7 (−25.2, 19.0)		29	−2.2 (−21.5, 16.2)		n.s.	<0.001 ^†^
	%^A→G^	30	61.8 (28.7, 149.5)		32	11.5 (−8.7, 26.1)		<0.001	30	19.5 (−8.0, 34.5)		28	6.1 (−6.0, 23.1)		n.s.	<0.001
C22:4*n*6	0	28	2.5 (2.1, 3.4)	a	30	2.9 (2.3, 3.5)	a	n.s.	30	3.1 (2.3, 3.5)	a	27	3.1 (2.7, 3.7)	a,b	n.s.	n.s.
	10		2.7 (±0.9) 2.6 (2.1, 3.1)	a,b		3.0 (2.6, 3.4)	a	n.s.		3.1 (±0.7) 3.0 (2.7, 3.5)	a		3.6 (3.0, 3.7)	a	n.s.	n.s. ^†^
	20		2.4 (±0.6) 2.4 (2.1, 2.9)	b		2.9 (±0.6) 3.0 (2.5, 3.3)	a	<0.001 ^†^		2.9 (±0.5) 2.8 (2.5, 3.2)	a		3.1 (2.7, 3.4)	b	n.s.	0.001 ^†^
	%^A→F^	28	−1.4 (−30.1, 8.5)		30	6.1 (−1.3, 23.1)		0.037	30	−3.0 (−9.8, 29.8)		29	3.9 (−8.1, 24.1)		n.s.	n.s.
	%^A→G^	30	−19.4 (−30.0, −3.2)		32	−0.9 (−10.6, 16.5)		0.001	30	−7.3 (−16.0, 17.1)		28	−2.4 (−14.9, 11.8)		n.s.	0.044
C22:5*n*6	0	28	0.4 (0.4, 0.9)	a	30	0.4 (0.3, 0.6)	a	n.s.	30	0.6 (0.4, 1.0)	a	27	0.8 (0.5, 1.1)	a,b	n.s.	n.s.
	10		0.8 (0.5, 1.1)	a		0.7 (0.5, 1.0)	b	n.s.		0.9 (0.7, 1.0)	a		0.9 (0.8, 1.1)	a	n.s.	n.s.
	20		0.6 (0.6, 0.7)	a		0.7 (0.5, 0.9)	b	n.s.		0.7 (±0.2) 0.7 (0.6, 0.8)	a		0.7 (±0.2) 0.7 (0.6, 0.8)	b	n.s. ^†^	n.s.
	%^A→F^	28	49.7 (−15.0, 150.3)		30	99.5 (2.6, 165.8)		n.s.	30	8.8 (−28.7, 109.1)		29	15.3 (−24.0, 93.8)		n.s.	n.s.
	%^A→G^	30	20.8 (−38.8, 59.7)		32	39.7 (−14.5, 127.0)		n.s.	30	−5.2 (−42.4, 66.4)		28	−11.2 (−45.8, 85.1)		n.s.	n.s.
C22:5*n*3 (DPA)	0	28	2.3 (2.0, 2.6)	a	30	2.2 (2.0, 2.7)	a	n.s.	30	2.3 (±0.7) 2.3 (2.0, 2.7)	a ^†^	27	2.5 (±0.6)	a ^†^	n.s. ^†^	n.s.
	10		3.4 (2.9, 3.9)	b		2.4 (2.1, 2.9)	a,b	<0.001		2.5 (±0.6) 2.6 (2.2, 2.9)	a,b ^†^		2.5 (±0.6)	a ^†^	n.s. ^†^	<0.001
	20		3.2 (±0.5) 3.1 (2.9, 3.5)	b		2.6 (±0.4) 2.6 (2.3, 2.8)	b	<0.001 ^†^		2.6 (±0.3)	b ^†^		2.7 (±0.3)	a ^†^	n.s. ^†^	<0.001 ^†^
	%^A→F^	28	53.4 (15.4, 94.5)		30	4.7 (−7.5, 15.8)		0.001	30	5.3 (−4.5, 35.1)		29	10.5 (−16.8, 20.1)		n.s.	0.003
	%^A→G^	30	34.6 (19.4, 58.8)		32	7.5 (−0.6, 20.1)		<0.001	30	16.1 (−0.6, 27.7)		28	11.6 (−6.5, 23.1)		n.s.	0.005
C22:6*n*3 (DHA)	0	28	3.8 (3.2, 4.4)	a	30	3.9 (±1.2) 3.7 (3.1, 4.7)	a ^†^	n.s.	30	3.9 (±1.0) 3.9 (3.3, 4.7)	a ^†^	26	4.0 (±0.9)	a ^†^	n.s. ^†^	n.s.
	10		5.0 (4.5, 5.7)	b		4.0 (±1.1) 4.0 (3.1, 4.8)	a ^†^	0.001		4.4 (±0.9) 4.5 (3.8, 4.8)	a,b ^†^		3.9 (±0.9)	a ^†^	n.s. ^†^	0.010
	20		4.9 (±0.8) 4.8 (4.4, 5.4)	b		4.1 (±0.9)	a ^†^	<0.001 ^†^		4.3 (±0.7)	b ^†^		4.3 (±0.7)	a ^†^	n.s. ^†^	0.004 ^†^
	%^A→F^	28	35.1 (±41.5) 37.4 (1.1, 66.4)		30	0.7 (−12.4, 15.7)		0.006	30	18.6 (±39.4)		28	0.3 (±25.9)		0.043 ^†^	n.s. ^†^
	%^A→G^	30	30.0 (±31.5) 25.8 (10.5, 40.1)		32	9.1 (±22.3)		0.003 ^†^	30	6.6 (−2.8, 21.1)		27	12.0 (−7.9, 20.4)		n.s.	0.010
*n*-3 index	0	28	4.7 (4.1, 5.4)	a	30	4.4 (3.8, 5.8)	a	n.s.	30	4.8 (±1.2) 4.7 (4.0, 5.5)	a ^†^	26	4.9 (±1.1)	a ^†^	n.s. ^†^	n.s.
	10		7.8 (±1.9) 7.8 (6.9, 8.8)	b		4.9 (±1.4) 5.2 (3.9, 5.9)	a	<0.001 ^†^		5.2 (±1.0)	a,b ^†^		4.7 (±1.1)	a ^†^	n.s. ^†^	<0.001 ^†^
	20		6.6 (±1.1) 6.7 (5.9, 7.3)	c		5.0 (4.4, 5.6)	a	<0.001		5.3 (±0.8)	b ^†^		5.3 (±1.1)	a ^†^	n.s. ^†^	<0.001 ^†^
	%^A→F^	28	64.6 (±45.0) 61.1 (37.6, 100.0)		30	−1.8 (−7.5, 18.6)		<0.001	30	15.1 (±35.0)		28	−0.5 (±22.4)		0.049 ^†^	<0.001 ^†^
	%^A→G^	30	39.2 (±32.3) 36.3 (16.1, 59.2)		32	9.5 (±22.4)		<0.001 ^†^	30	6.3 (−1.3, 22.5)		27	10.9 (−6.0, 21.8)		n.s.	<0.001
SFA	0	27	34.9 (33.1, 38.2)	a	28	34.4 (33.6, 35.4)	a	n.s.	29	33.9 (32.5, 35.3)	a	27	34.0 (32.8, 36.9)	a	n.s.	n.s.
	10		35.1 (33.8, 38.1)	a		35.3 (33.8, 36.9)	a	n.s.		33.5 (33.1, 34.9)	a		34.4 (33.5, 37.3)	a	n.s.	0.037
	20		34.0 (33.2, 35.4)	a		34.5 (33.8, 36.3)	a	n.s.		34.1 (±1.2) 33.8 (33.4, 34.9)	a		34.2 (±1.3) 34.3 (33.4, 35.1)	a	n.s. ^†^	n.s.
	%^A→F^	27	1.7 (±18.3)		28	3.1 (±11.1)		n.s. ^†^	29	−1.0 (±19.0) −0.4 (−6.5, 4.8)		29	2.4 (−6.2, 12.5)		n.s.	n.s. ^†^
	%^A→G^	29	−0.4 (−13.73 7.5)		30	1.5 (−2.5, 7.6)		n.s.	29	0.7 (−4.2, 5.5)		28	1.2 (−10.6, 4.0)		n.s.	n.s.
MUFA	0	27	17.1 (16.3, 17.7)	a	30	17.0 (16.3, 17.5)	a	n.s.	30	16.3 (15.7, 17.6)	a	27	16.2 (15.4, 17.1)	a	n.s.	n.s.
	10		17.0 (16.2, 17.8)	a		17.0 (16.3, 18.1)	a	n.s.		17.8 (17.2, 18.5)	b		17.2 (16.1, 17.9)	b	n.s.	0.008
	20		17.3 (±1.5) 17.5 (16.4, 18.5)	a		17.4 (15.5, 18.0)	a	n.s.		17.3 (±1.2) 17.3 (16.8, 18.0)	a,b		17.1 (±1.0) 17.2 (16.5, 17.7)	a,b	n.s. ^†^	n.s. ^†^
	%^A→F^	27	−0.4 (−8.3, 5.0)		30	0.6 (−5.5, 4.1)		n.s.	30	8.8 (1.3, 16.5)		29	5.0 (0.2, 10.7)		n.s.	0.002
	%^A→G^	29	2.5 (−2.7, 9.1)		32	0.0 (−5.0, 5.1)		n.s.	30	1.8 (−0.9, 8.7)		28	2.6 (−1.2, 12.1)		n.s.	n.s.
C18:1c9/C18:0	0	27	1.3 (±0.3) 1.2 (1.1, 1.4)	a	30	1.3 (1.1, 1.4)	a	n.s.	30	1.2 (±0.4)	a ^†^	27	1.1 (±0.3) 1.1 (0.9, 1.4)	a	n.s. ^†^	n.s. ^†^
	10		1.2 (1.1, 1.4)	a		1.2 (1.1, 1.3)	a	n.s.		1.5 (±0.2) 1.4 (1.3, 1.6)	b ^†^		1.3 (1.2, 1.4)	a	0.005	<0.001
	20		1.3 (±0.3) 1.3 (1.2, 1.5)	a		1.3 (1.0, 1.4)	a	n.s.		1.3 (±0.2)	a ^†^		1.2 (±0.2) 1.2 (1.1, 1.4)	a	n.s. ^†^	n.s. ^†^
	%^A→F^	27	−8.8 (−22.4, 16.0)		30	−8.1 (−17.1, 9.2)		n.s.	30	16.8 (2.8, 39.5)		29	17.3 (−6.5, 32.6)		n.s.	0.004
	%^A→G^	29	2.4 (−16.0, 34.9)		32	−2.9 (−18.2, 22.6)		n.s.	30	−1.0 (−12.3, 29.2)		28	6.9 (−6.7, 20.8)		n.s.	n.s.
PUFA	0	26	37.6 (36.7, 38.7)	a	28	37.8 (36.3, 38.8)	a	n.s.	29	38.8 (36.9, 39.2)	a	26	38.4 (36.2, 39.6)	a	n.s.	n.s.
	10		37.6 (36.0, 38.7)	a		37.1 (35.3, 38.9)	a	n.s.		38.3 (37.4, 39.1)	a		38.1 (36.4, 39.7)	a	n.s.	n.s.
	20		38.1 (36.8, 38.5)	a		37.7 (36.9, 38.4)	a	n.s.		38.1 (±1.0) 38.1 (37.4, 38.9)	a		38.4 (±1.0) 38.5 (37.9, 38.7)	a	n.s. ^†^	n.s.
	%^A→F^	26	0.2 (−8.8, 5.4)		28	1.0 (−5.7, 5.3)		n.s.	29	1.0 (−3.0, 3.7)		28	1.2 (−6.9, 3.6)		n.s.	n.s.
	%^A→G^	29	1.1 (−3.7, 4.8)		31	−0.1 (−4.0, 2.5)		n.s.	30	−0.5 (−2.4, 2.6)		27	0.3 (−1.7, 6.4)		n.s.	n.s.
*n*-6 PUFA	0	28	29.3 (±2.9) 29.9 (27.7, 31.0)	a ^†^	29	29.0 (±2.9)	a ^†^	n.s. ^†^	29	30.7 (29.4, 31.5)	a	27	30.3 (28.1, 31.4)	a	n.s.	n.s.
	10		25.1 (±2.7) 25.2 (23.5, 26.3)	b ^†^		28.7 (±2.2)	a ^†^	<0.001 ^†^		29.6 (28.3, 30.1)	a		30.1 (29.1, 31.2)	a	n.s.	<0.001
	20		27.3 (±1.8)	c ^†^		28.6 (±2.5)	a ^†^	0.032 ^†^		29.6 (±1.0) 29.8 (29.3, 30.4)	a		30.2 (29.4, 30.8)	a	n.s.	<0.001 ^†^
	%^A→F^	28	−15.0 (−21.1, −9.9)		29	1.0 (−7.8, 4.1)		<0.001	29	−2.5 (−6.6, 1.7)		29	−0.8 (−7.9, 3.7)		n.s.	<0.001
	%^A→G^	30	−6.4 (−9.1, −2.0)		32	−2.2 (−6.7, 1.6)		n.s.	30	−1.7 (−5.0, 1.2)		28	−0.6 (−2.9, 4.5)		n.s.	0.004
*n*-3 PUFA	0	26	7.7 (6.9, 8.8)	a	28	7.7 (6.8, 8.7)	a	n.s.	30	7.6 (±1.6) 7.4 (6.8, 8.4)	a	26	7.9 (±1.5)	a ^†^	n.s. ^†^	n.s.
	10		12.0 (11.0, 13.0)	b		8.1 (6.8, 9.1)	a	<0.001		8.7 (7.7, 9.2)	b		7.8 (±1.6) 7.9 (7.3, 8.7)	a ^†^	0.040	<0.001
	20		10.5 (±1.4) 10.6 (9.6, 11.6)	c		8.1 (7.4, 8.9)	a	<0.001		8.5 (±0.9) 8.5 (7.9, 9.2)	b		8.4 (±1.2)	a ^†^	n.s. ^†^	<0.001 ^†^
	%^A→F^	26	57.0 (23.6, 88.3)		28	−1.2 (−10.0, 10.5)		<0.001	30	12.3 (−0.5, 22.7)		28	4.9 (−12.7, 16.0)		n.s.	<0.001
	%^A→G^	29	35.1 (15.2, 51.8)		31	6.6 (−2.8, 20.7)		<0.001	30	9.5 (0.2, 15.9)		27	12.4 (−5.5, 17.8)		n.s.	<0.001
*n*-6/*n*-3 PUFA	0	26	3.8 (±0.9) 3.9 (3.3, 4.3)	a	28	3.8 (±0.8)	a ^†^	n.s. ^†^	29	3.9 (±0.6) 3.9 (3.5, 4.3)	a	26	3.8 (±0.7)	a ^†^	n.s. ^†^	n.s. ^†^
	10		2.1 (±0.4) 2.0 (1.9, 2.4)	b		3.8 (±0.9)	a,b ^†^	<0.001 ^†^		3.5 (3.1, 3.8)	b		3.8 (±0.7) 3.7 (3.4, 4.1)	a ^†^	n.s.	<0.001
	20		2.5 (2.3, 3.0)	c		3.5 (±0.7) 3.4 (3.2, 3.9)	b ^†^	<0.001		3.5 (±0.4) 3.6 (3.1, 3.9)	b		3.7 (±0.7)	a ^†^	n.s. ^†^	<0.001
	%^A→F^	26	−42.1 (±16.2)		28	0.2 (±20.9)		<0.001 ^†^	29	−6.8 (±16.5) −9.7 (−18.6, 1.2)		28	−4.2 (−9.2, 13.3)		n.s.	<0.001 ^†^
	%^A→G^	29	−29.1 (−38.2, −18.5)		31	−6.9 (−15.9, 2.8)		<0.001	30	−7.2 (±10.9) −7.7 (−12.5, 1.5)		27	−4.0 (±15.7)		n.s. ^†^	<0.001
LA/ALA	0	28	56.8 (±21.3)	a ^†^	30	49.7 (±14.7)	a ^†^	n.s. ^†^	30	55.2 (±18.5) 52.9 (41.9, 67.4)	a	27	58.5 (±19.7)	a ^†^	n.s. ^†^	n.s. ^†^
	10		50.1 (±17.9) 47.5 (39.0, 61.4)	a ^†^		49.4 (±11.1)	a ^†^	n.s. ^†^		37.8 (33.6, 46.7)	b		57.6 (±20.0) 58.3 (49.6, 70.3)	a ^†^	<0.001	0.026
	20		61.1 (±21.2) 59.3 (44.2, 78.7)	a ^†^		57.2 (±11.0)	b ^†^	n.s. ^†^		46.8 (40.7, 53.9)	a,b		61.7 (±17.4) 62.4 (49.3, 72.6)	a ^†^	0.001	0.008
	%^A→F^	28	−6.7 (−38.6, 24.5)		30	−6.0 (−19.6, 35.5)		n.s.	30	−28.7 (−38.2, −6.6)		29	−9.0 (−17.8, 22.1)		0.003	n.s.
	%^A→G^	30	−0.2 (−22.1, 49.6)		32	5.7 (−8.9, 44.8)		n.s.	30	−18.9 (−27.9, 18.8)		28	6.8 (−22.6, 27.6)		n.s.	0.046
TFA	0	28	0.6 (0.5, 0.8)	a	30	0.6 (0.5, 0.7)	a	n.s.	30	0.5 (0.5, 0.6)	a	27	0.5 (0.5, 0.6)	a	n.s.	0.040
	10		0.6 (±0.1) 0.5 (0.5, 0.6)	b		0.5 (0.5, 0.6)	a	n.s.		0.6 (±0.2) 0.6 (0.5, 0.7)	a		0.5 (0.5, 0.6)	a	n.s.	n.s. ^†^
	20		0.6 (0.5, 0.7)	a		0.6 (0.5, 0.7)	a	n.s.		0.6 (0.5, 0.6)	a		0.6 (0.5, 0.7)	a	n.s.	n.s.
	%^A→F^	28	−13.1 (±33.8)		30	4.0 (±37.1)		n.s. ^†^	30	11.6 (±33.1)		29	3.9 (±32.8)		n.s. ^†^	0.007 ^†^
	%^A→G^	30	−2.6 (±43.3) −8.4 (−37.9, 17.0)		32	11.3 (−8.9, 31.7)		n.s.	30	6.9 (±23.3) 10.6 (−10.3, 19.3)		28	16.5 (−5.8, 30.0)		n.s.	n.s. ^†^
EPA/ALA	0	28	4.7 (3.6, 6.3)	a	30	4.4 (2.8, 6.4)	a	n.s.	30	4.3 (3.32 5.5)	a	27	3.9 (3.4, 5.1)	a,b	n.s.	n.s.
	10		14.9 (12.0, 18.9)	b		4.2 (3.5, 5.6)	a	<0.001		2.9 (2.3, 3.3)	b		3.9 (3.2, 4.8)	a	<0.001	<0.001
	20		8.4 (6.9, 10.1)	c		5.8 (4.5, 7.0)	b	<0.001		4.0 (3.2, 5.0)	a		4.9 (3.4, 6.0)	b	n.s.	<0.001
	%^A→F^	28	222.8 (141.2, 307.3)		30	−3.2 (−22.8, 22.6)		<0.001	30	−31.2 (−49.8, −23.4)		29	−13.7 (−23.9, 1.6)		<0.001	<0.001
	%^A→G^	30	75.7 (32.8, 157.1)		32	19.7 (−4.6, 56.6)		<0.001	30	−2.8 (−32.9, 16.4)		28	10.2 (−12.0, 35.8)		0.048	<0.001
DPA/ALA	0	28	13.0 (9.0, 16.0)	a	30	12.4 (8.6, 16.5)	a	n.s.	30	11.7 (9.0, 17.1)	a	27	14.6 (±6.9) 14.1 (9.9, 17.1)	a ^†^	n.s.	n.s.
	10		21.3 (±9.6) 18.6 (15.3, 26.8)	b		12.9 (9.9, 14.8)	a	<0.001		9.5 (±3.8) 9.1 (6.3, 11.4)	b		13.5 (±5.5)	a ^†^	0.003 ^†^	<0.001 ^†^
	20		19.1 (±8.4) 16.9 (12.7, 23.2)	b		15.1 (±3.9) 14.8 (13.6, 17.2)	a	0.025 ^†^		11.9 (±3.6) 12.0 (9.4, 14.2)	a,b		15.2 (±4.5)	a ^†^	0.003 ^†^	<0.001 ^†^
	%^A→F^	28	38.5 (14.9, 134.9)		30	0.6 (−25.7, 55.4)		0.007	30	−30.4 (−51.7, −0.4)		29	−4.9 (−29.0, 24.4)		0.035	<0.001
	%^A→G^	30	44.1 (2.1, 115.9)		32	14.3 (−11.6, 73.1)		n.s.	30	−13.3 (−31.4, 41.3)		28	2.6 (−9.1, 37.7)		n.s.	0.002
DHA/ALA	0	28	22.6 (14.2, 28.9)	a	30	19.1 (13.1, 25.8)	a	n.s.	30	23.0 (±10.5) 22.0 (13.1, 30.9)	a ^†^	26	22.4 (±8.9)	a ^†^	n.s. ^†^	n.s.
	10		30.8 (±12.3) 28.2 (22.5, 35.6)	b		20.4 (±7.6) 18.9 (15.6, 24.4)	a	<0.001 ^†^		16.4 (±6.6)	b ^†^		20.6 (±8.7)	a ^†^	0.047 ^†^	<0.001 ^†^
	20		29.4 (±12.1) 27.2 (22.4, 35.6)	b		23.8 (±6.4) 24.6 (19.9, 28.1)	a	0.035 ^†^		19.9 (±7.2)	a ^†^		24.5 (±9.0)	a ^†^	0.036 ^†^	<0.001 ^†^
	%^A→F^	28	36.2 (−2.1, 98.5)		30	−8.4 (−27.7, 38.3)		0.023	30	−24.8 (−51.1, −9.9)		28	−4.0 (−18.1, 12.6)		0.031	<0.001
	%^A→G^	30	35.6 (4.1, 84.8)		32	12.2 (−10.1, 60.9)		n.s.	30	−15.3 (−30.4, 26.1)		27	5.7 (−6.5, 27.8)		n.s.	0.004
ARA/LA	0	28	1.3 (±0.2)	a ^†^	30	1.4 (±0.3)	a ^†^	n.s. ^†^	30	1.3 (±0.3)	a ^†^	27	1.3 (±0.3)	a ^†^	n.s. ^†^	n.s. ^†^
	10		1.4 (±0.3)	a ^†^		1.3 (±0.3)	b ^†^	n.s. ^†^		1.1 (±0.2)	b ^†^		1.1 (±0.2)	b ^†^	n.s. ^†^	<0.001 ^†^
	20		1.2 (±0.2)	b ^†^		1.3 (±0.2)	b ^†^	n.s. ^†^		1.2 (±0.2)	c ^†^		1.3 (±0.2)	a ^†^	n.s. ^†^	n.s. ^†^
	%^A→F^	28	6.8 (±23.6)		30	−6.2 (±17.5)		0.020 ^†^	30	−17.3 (±21.5)		29	−9.8 (±19.7)		n.s. ^†^	<0.001 ^†^
	%^A→G^	30	−8.7 (±20.3)		32	−7.9 (±20.4)		n.s. ^†^	30	−5.0 (±18.5)		28	1.2 (±19.4)		n.s. ^†^	n.s. ^†^
ARA/EPA	0	28	15.9 (11.6, 19.8)	a	30	15.0 (12.9, 19.2)	a	n.s.	30	15.9 (13.8, 18.1)	a	27	17.6 (±7.0) 16.8 (12.3, 22.3)	a ^†^	n.s.	n.s.
	10		4.1 (3.4, 5.1)	b		15.7 (11.6, 18.0)	a	<0.001		14.8 (12.6, 17.1)	a		16.5 (±6.5) 15.7 (12.5, 19.9)	a ^†^	n.s.	<0.001
	20		8.1 (5.8, 11.1)	c		13.1 (10.8, 16.0)	a	<0.001		15.0 (±4.4) 14.7 (11.7, 17.3)	a		16.8 (±7.0)	a ^†^	n.s. ^†^	<0.001
	%^A→F^	28	−73.7 (−77.2, −61.7)		30	1.7 (−21.7, 21.6)		<0.001	30	−5.0 (−23.1, 12.0)		29	−7.2 (−15.8, 10.3)		n.s.	<0.001
	%^A→G^	30	−41.5 (±24.8)		32	−10.7 (±25.5)		<0.001 ^†^	30	−4.3 (±30.7)		28	−2.0 (±32.5)		n.s. ^†^	<0.001 ^†^
ARA/DHA	0	28	3.5 (±1.0)	a ^†^	30	3.7 (±1.0) 3.7 (3.0, 4.3)	a	n.s. ^†^	30	3.4 (±0.7)	a ^†^	26	3.3 (±0.8)	a ^†^	n.s. ^†^	n.s. ^†^
	10		2.3 (±0.7)	b ^†^		3.4 (±1.2) 3.2 (2.5, 4.0)	a,b	<0.001 ^†^		2.8 (±0.5)	b ^†^		3.1 (±0.7)	a ^†^	0.035 ^†^	0.004 ^†^
	20		2.5 (±0.5) 2.4 (2.2, 2.7)	b ^†^		3.3 (2.4, 3.5)	b	0.004		3.1 (±0.4)	c ^†^		3.2 (±0.8)	a ^†^	n.s. ^†^	<0.001 ^†^
	%^A→F^	28	−34.7 (−45.3, −19.1)		30	−3.5 (−20.1, 4.9)		<0.001	30	−16.2 (−28.2, −4.3)		28	−6.2 (−20.3, 1.0)		n.s.	0.007
	%^A→G^	30	−28.3 (−38.8, −14.6)		32	−13.7 (−20.4, −3.6)		0.003	30	−6.9 (−13.5, −1.4)		27	−3.4 (−14.6, 10.1)		n.s.	<0.001

* Variables expressed as mean (±SD) and/or as median (25th, 75th percentile) depending on the statistical test that was performed; ∆ Differences within groups comparing points in time, points in time without a common letter are significantly different, *p* < 0.05; ◊ Differences between each intervention group and their corresponding control group; ● Differences between both intervention groups; %^A→F^, percentage change from baseline to week 10; %^A→G^, percentage change from baseline to follow-up; ^†^ Calculated with parametric test. Abbreviations: ARA, arachidonic acid; ALA, *α*-linolenic acid; CLA, conjugated linoleic acid; DGLA, dihomo-*γ*-linolenic acid; DHA, docosahexaenoic acid; DPA, docosapentaenoic acid; EPA, eicosapentaenoic acid; ETA, eicosatetraenoic acid; FAME, fatty acid methyl ester; HTGC, hypertriglyceridemia control; HTGI, hypertriglyceridemia intervention; LA, linoleic acid; MUFA, monounsaturated fatty acids; PDC, prediabetes control; PDI, prediabetes intervention; PUFA, polyunsaturated fatty acids; SFA, saturated fatty acids; TFA, trans-fatty acids.

## Data Availability

The original contributions presented in the study are included in the article/Supplementary Material, further inquiries can be directed to the corresponding author due to privacy.

## Supplementary Materials

The following supporting information can be downloaded at: https://www.mdpi.com/article/10.3390/nu16091261/s1, Table S1: Biochemical methods; Table S2: Daily energy and macronutrient intake of the study subjects in each group before baseline assessment (full self-reports, 5 days); Table S3: Nutrient status in blood at baseline, after the intervention period and at follow-up; Table S4: Nutrient status in 24 h urine at baseline, after the intervention period and at follow-up.
