# Supplementary material for: Validation of Nutritional Approaches to Modulate Cardiovascular and Diabetic Risk Factors in Patients with Hypertriglyceridemia or Prediabetes—The MoKaRi II Randomized Controlled Study
Source: Nutrients. 2024 Apr 24;16(9):1261. doi: 10.3390/nu16091261 (PMC11085300; doi:10.3390/nu16091261)
Supplement: Supplementary file 1 [file nutrients-16-01261-s001.zip › nutrients-2979840-supplementary.pdf]

Supplementary Materials

Table S1. Biochemical methods.

Parameter	Instrument	Reference range
	Plasma, serum	
Total cholesterol [mmol/L]*	Cobas 8000 (Roche, Mannheim, Germany)	< 5.2
LDL cholesterol [mmol/L]*	Cobas 8000 (Roche, Mannheim, Germany)	< 3.35
HDL cholesterol [mmol/L]*	Cobas 8000 (Roche, Mannheim, Germany)	> 1.03
Triglycerides [mmol/L]*	Cobas 8000 (Roche, Mannheim, Germany)	< 1.7
High-sensitivity CRP [mg/L]*	Cobas 8000 (Roche, Mannheim, Germany)	≤ 0.3
Blood glucose [mmol/L]*	Cobas 8000 (Roche, Mannheim, Germany)	18 – 60 y: 4.1 – 5.9<br>60 – 90 y: 4.6 – 6.4
Insulin [mU/L]*	Cobas 8000 (Roche, Mannheim, Germany)	3 – 25
C-peptide [ng/mL]*	Cobas 8000 (Roche, Mannheim, Germany)	1.1 – 4.4
HbA1c [%]*	Tosoh HLC-723G11 (Sysmex, Norderstedt, Germany)	4.5 – 6.1
GGT [μmol/l*s]*	Cobas 8000 (Roche, Mannheim, Germany)	0.17 – 1.19
Folic acid [ng/mL]*	Cobas 8000 (Roche, Mannheim, Germany)	No information available
Vitamin B_{12} [pmol/L]*	Cobas 8000 (Roche, Mannheim, Germany)	197 – 7712
Holo-Transcobalamine [pmol/L]*	Cobas 8000 (Roche, Mannheim, Germany)	> 37.52
Vitamin B_1 [nmol/L]*	HPLC (Shimadzu, Kyoto, Japan)	47 – 1412
Vitamin B_6 [nmol/L]*	HPLC (Shimadzu, Kyoto, Japan)	14.6 – 72.8
Vitamin A [μmol/L]*	HPLC (Shimadzu, Kyoto, Japan)	No information available
Vitamin D [nmol/L]*	Cobas 8000 (Roche, Mannheim, Germany)	> 75
Vitamin E [μmol/L]*	HPLC (Shimadzu, Kyoto, Japan)	11.6 – 46.4
Ferritin [μg/L]*	Cobas 8000 (Roche, Mannheim, Germany)	Women: 13 – 150<br>Men: 30 – 400
Transferrin [g/L]*	Cobas 8000 (Roche, Mannheim, Germany)	2.0 – 3.6

Table S1. Continued.

Parameter	Instrument	Reference range
	Urine	
Calcium 24h urine [mmol/24h]*	Cobas 8000 (Roche, Mannheim, Germany)	1.3 – 7.5
Chloride 24h urine [mmol/24h]*	Cobas 8000 (Roche, Mannheim, Germany)	110 – 250
Potassium 24h urine [mmol/24h]*	Cobas 8000 (Roche, Mannheim, Germany)	44 – 90
Creatinine 24h urine [mmol/24h]*	Cobas 8000 (Roche, Mannheim, Germany)	8.0 – 26.5
Magnesium 24h urine [mmol/24h]*	Cobas 8000 (Roche, Mannheim, Germany)	No information available
Sodium 24h urine [mmol/24h]*	Cobas 8000 (Roche, Mannheim, Germany)	94 – 222
Selenium 24h urine [µmol/24h]*	AAS 5 FL (Analytik Jena AG, Jena, Germany)	No information available
Zinc 24h urine [µmol/24h]*	AAS 5 FL (Analytik Jena AG, Jena, Germany)	No information available
	Erythrocytes	
Fatty acids [% FAME]◊	GC-17V3 (Shimadzu, Duisburg, Germany)	No information available

* Measured by Institute of Clinical Chemistry and Laboratory Diagnostics, University Hospital Jena, Jena, Germany; ◊ Measured by Institute of Nutritional Sciences, Friedrich Schiller University, Jena, Germany. Abbreviations: C-peptide, connecting peptide; FAME, fatty acid methyl ester; GGT, gamma glutamyl transferase; HbA1c, glycated hemoglobin A_{1c}; HDL, high-density lipoprotein; CRP, c-reactive protein; LDL, low-density lipoprotein.

Table S2. Daily energy and macronutrient intake of the study subjects in each group before baseline assessment (full self-reports, 5 days).

**Energy and nutrients**	**DGE reference values 51 to 64 years**	**HTGI (*n* = 30) Characteristics***	**HTGC (*n* = 33) Characteristics***	◊	**PDI (*n* = 30) Characteristics***	**PDC (*n* = 31) Characteristics***	◊	●
Energy [kcal/day]	w: 1700<br>m: 2200	2307 (± 589)	2254 (± 559)	n.s.†	2364 (± 430)	2223 (± 483)	n.s.†	n.s.†
Carbohydrate [g/day]	> 50 en%	217 (196, 273)	213 (183, 258)	n.s.	245 (± 42)<br>246 (214, 280)	229 (± 61)	n.s.†	n.s.
Fiber [g/day]	≥ 30	24.4 (19.8, 31.6)	21.9 (15.6, 28.4)	n.s.	27.5 (25.0, 31.1)	25.3 (20.3, 31.1)	n.s.	n.s.
Total sugar [g/day]	n.a.	90 (73, 125)	92 (78, 120)	n.s.	117 (95, 128)	104 (85, 120)	n.s.	0.049
Sucrose [g/day]	n.a.	45.8 (± 22.4)<br>43.2 (31.5, 56.3)	43.8 (30.4, 52.7)	n.s.	49.4 (± 15.2)<br>49.8 (38.2, 60.8)	52.2 (42.1, 61.5)	n.s.	n.s.†
Glucose [g/day]	n.a.	15.4 (12.2, 21.7)	19.0 (14.3, 26.0)	n.s.	21.4 (± 6.6)<br>20.9 (16.4, 25.2)	19.6 (± 7.0)	n.s.†	0.011
Fructose [g/day]	n.a.	18.9 (13.9, 25.6)	24.4 (16.9, 31.0)	n.s.	27.3 (± 9.1)<br>26.6 (21.0, 31.9)	23.6 (± 9.4)	n.s.†	0.002
Alcohol [g/day]	n.a.	1.9 (0.2, 13.5)	10.9 (3.9, 23.2)	0.006	4.7 (2.5, 10.3)	3.5 (0.2, 10.9)	n.s.	n.s.
Protein [g/day]	0.8 g/kg body weight	90 (± 28)<br>86 (70, 101)	88 (± 22)	n.s.†	89 (79, 107)	84 (68, 100)	n.s.	n.s.
Fat [g/day]	30 en%	97 (71, 120)	91 (75, 106)	n.s.	98 (± 27)<br>96 (81, 123)	93 (± 25)	n.s.†	n.s.
SFA [g/day]	< 10 en%	40.3 (± 13.1)	36.3 (± 12.5)	n.s.†	41.4 (± 13.8)	37.3 (± 9.3)	n.s.†	n.s.†
MUFA [g/day]	> 10 en%	32.8 (± 10.4)<br>32.4 (24.3, 40.6)	31.6 (24.8, 37.5)	n.s.	34.1 (± 9.2)	32.6 (± 10.2)	n.s.†	n.s.†
PUFA [g/day]	7-10 en%	12.6 (10.4, 17.1)	12.3 (10.2, 16.4)	n.s.	13.7 (11.1, 18.4)	15.2 (12.0, 19.1)	n.s.	n.s.
C-18:2n6 [g/day]	2.5 en%	10.3 (8.5, 13.0)	10.0 (8.2, 13.7)	n.s.	10.6 (8.6, 12.8)	12.3 (10.0, 15.8)	n.s.	n.s.

Table S2. Continued.

Energy and nutrients	DGE reference values 51 to 64 years	HTGI (*n* = 30) Characteristics*	HTGC (*n* = 33) Characteristics*	◊	PDI (*n* = 30) Characteristics*	PDC (*n* = 31) Characteristics*	◊	●
C-18:3n3 [g/day]	0.5 en%	1.5 (0.9, 2.5)	1.4 (1.0, 1.9)	n.s.	1.7 (1.3, 2.2)	1.7 (1.2, 2.4)	n.s.	n.s.
C-20:5n3 [g/day]	0.25	0.1 (0.0, 0.4)	0.1 (0.0, 0.4)	n.s.	0.1 (0.0, 0.4)	0.1 (0.0, 0.2)	n.s.	n.s.
C-22:6n3 [g/day]		0.2 (0.1, 0.4)	0.2 (0.0, 0.4)	n.s.	0.3 (0.1, 0.5)	0.2 (0.1, 0.3)	n.s.	n.s.
C-20:4n6 [g/day]	n.a.	0.2 (0.1, 0.2)	0.2 (0.1, 0.3)	n.s.	0.2 (0.1, 0.3)	0.2 (0.1, 0.3)	n.s.	n.s.
Cholesterol [mg/day]	< 300	363 (± 162)<br>340 (253, 471)	380 (± 175)	n.s.†	369 (274, 510)	315 (256, 414)	n.s.	n.s.
Vitamin A [mg/day]	w: 0.70<br>m: 0.85	0.6 (0.4, 1.1)	0.5 (0.3, 0.6)	n.s.	0.6 (0.4, 1.0)	0.5 (0.4, 0.8)	n.s.	n.s.
Vitamin B_1 [mg/day]	w: 1.0<br>m: 1.2	1.6 (± 0.6)	1.6 (± 0.6)	n.s.†	1.6 (± 0.4)	1.6 (± 0.6)	n.s.†	n.s.†
Vitamin B_2 [mg/day]	w: 1.0<br>m: 1.3	1.6 (1.3, 2.1)	1.5 (1.4, 1.9)	n.s.	1.8 (1.5, 2.1)	1.6 (1.3, 2.0)	n.s.	n.s.
Vitamin B_6 [mg/day]	w: 1.4<br>m: 1.6	2.0 (± 0.7)	2.0 (± 0.6)	n.s.†	2.1 (± 0.5)	1.9 (± 0.5)	n.s.†	n.s.†
Vitamin B_{12} [µg/day]	4	5.6 (3.8, 7.3)	5.3 (3.6, 7.6)	n.s.	6.1 (4.5, 8.5)	4.5 (3.5, 6.1)	n.s.	n.s.
Vitamin C [mg/day]	w: 95<br>m: 110	128 (75, 220)	115 (72, 175)	n.s.	157 (126, 188)	129 (106, 174)	n.s.	n.s.
Vitamin D [µg/day]	20	2.9 (1.3, 5.3)	2.8 (1.8, 6.2)	n.s.	2.8 (1.7, 4.8)	2.5 (1.4, 3.6)	n.s.	n.s.
Vitamin E [mg/day]	w: 12<br>m: 13	10.3 (8.1, 15.4)	11.0 (8.8, 15.7)	n.s.	11.6 (± 3.2)<br>11.5 (9.7, 12.9)	12.8 (± 5.1)	n.s.†	n.s.
Vitamin K [µg/day]	w: 65<br>m: 80	142 (95, 286)	143 (82, 227)	n.s.	144 (98, 273)	128 (78, 182)	n.s.	n.s.

Table S2. Continued.

**Energy and nutrients**	**DGE reference values 51 to 64 years**	**HTGI (*n* = 30) Characteristics***	**HTGC (*n* = 33) Characteristics***	◊	**PDI (*n* = 30) Characteristics***	**PDC (*n* = 31) Characteristics***	◊	●
Calcium [mg/day]	1000	839 (626, 1171)	816 (693, 964)	n.s.	993 (676, 1157)	807 (655, 1078)	n.s.	n.s.
Magnesium [mg/day]	w: 300<br>m: 350	373 (287, 492)	373 (329, 440)	n.s.	394 (341, 457)	379 (324, 452)	n.s.	n.s.
Potassium [mg/day]	4000	3526 (2718, 4286)	3464 (2663, 4302)	n.s.	3755 (± 615)<br>3832 (3467, 4061)	3547 (± 1041)	n.s.†	n.s.
Iron [mg/day]	10	12.8 (10.1, 16.2)	12.2 (10.8, 15.9)	n.s.	13.8 (± 1.8)<br>13.6 (12.4, 15.1)	13.6 (± 4.4)	n.s.†	n.s.
Zinc [mg/day]	w: 10<br>m: 16	10.9 (8.3, 15.6)	11.1 (9.1, 12.5)	n.s.	12.5 (± 2.6)<br>12.5 (10.6, 14.5)	11.5 (± 2.9)	n.s.†	n.s.
Sodium [mg/day]	1500	2622 (1799, 3291)	2320 (1894, 2695)	n.s.	2699 (2099, 3191)	2354 (1984, 2745)	n.s.	n.s.
Chloride [mg/day]	2300	4128 (2831, 5140)	3741 (3122, 4464)	n.s.	4314 (± 1530)<br>4096 (3312, 4852)	3842 (± 1091)	n.s.†	n.s.
Phosphor [mg/day]	700	1356 (1138, 1825)	1413 (1220, 1625)	n.s.	1581 (± 322)<br>1579 (1367, 1735)	1433 (± 427)	n.s.†	n.s.
Iodine [µg/day]	180	108 (77, 128)	95 (75, 137)	n.s.	118 (± 42)<br>119 (83, 147)	105 (± 49)	n.s.†	n.s.
Copper [µg/day]	1000-1500	2042 (± 775)<br>1878 (1510, 2356)	1954 (1729, 2273)	n.s.	2031 (± 386)	2197 (± 653)	n.s.†	n.s.†
Manganese [µg/day]	2000-5000	4264 (3459, 5524)	4328 (3484, 5421)	n.s.	4960 (4096, 5809)	4527 (3435, 6133)	n.s.	n.s.

* Variables expressed as mean (± SD) and/or as median (25th, 75th percentile) depending on the statistical test that was performed; ◊ Differences between each intervention group and their corresponding control group; ● Differences between both intervention groups; † Calculated with parametric test. Abbreviations: DGE, German Nutrition Society; en%, percent of daily energy intake; HTGC, hypertriglyceridemia control; HTGI, hypertriglyceridemia intervention; MUFA, monounsaturated fatty acids; PDC, prediabetes control; PDI, prediabetes intervention; PUFA, polyunsaturated fatty acids; SFA, saturated fatty acids.

Table S3. Nutrient status in blood at baseline, after the intervention period and at follow-up.

Parameters	Week	HTGI *n*	HTGI Characteristics*	HTGI Δ	HTGC *n*	HTGC Characteristics*	HTGC Δ	◊	PDI *n*	PDI Characteristics*	PDI Δ	PDC *n*	PDC Characteristics*	PDC Δ	◊	●
Folic acid [ng/mL]	0		9.5 (7.3, 13.3)	a		10.0 (6.3, 13.3)	a	n.s.		7.8 (6.2, 9.7)	a		9.5 (6.0, 11.7)	a	n.s.	0.049
	10	29	9.7 (7.1, 11.5)	a	33	8.5 (6.1, 13.4)	a	n.s.	30	9.0 (7.3, 10.5)	b	29	7.4 (5.5, 11.5)	a	n.s.	n.s.
	20		10.0 (7.0, 11.6)	a		8.8 (5.3, 13.7)	a	n.s.		8.1 (6.3, 10.9)	a,b		9.0 (5.1, 12.2)	a	n.s.	n.s.
	$\%^{A\rightarrow F}$	29	2.4 (± 28.9)		33	-5.5 (± 23.1)		n.s.†	30	19.1 (± 33.1)<br>14.6 (-0.4, 37.3)		31	-8.5 (-23.2, 0.6)		0.001	0.044†
	$\%^{A\rightarrow G}$	30	-2.6 (± 31.8)		33	-6.6 (± 31.5)		n.s.†	30	12.7 (± 36.1)		29	-5.6 (± 25.2)		0.028†	n.s.†
Vitamin B_{12} [pmol/L]	0		256.0 (199.0, 326.0)	a		269.0 (207.0, 336.0)	a	n.s.		288.0 (245.0, 337.5)	a		318.0 (269.0, 400.0)	a	n.s.	n.s.
	10	29	220.0 (175.0, 284.0)	b	33	234.0 (184.0, 307.0)	b	n.s.	30	237.5 (± 73.6)<br>229.0 (177.8, 288.5)	b	29	312.3 (± 115.3)<br>283.0 (244.0, 362.0)	b	0.005†	n.s.
	20		267.0 (194.0, 298.0)	a,b		245.0 (205.0, 313.0)	a,b	n.s.		236.0 (171.8, 341.5)	a		297.0 (253.0, 385.0)	a,b	n.s.	n.s.
	$\%^{A\rightarrow F}$	29	-9.1 (± 18.7)		33	-9.0 (± 12.7)		n.s.†	30	-16.6 (± 15.9)		31	-8.2 (± 15.0)		0.036†	n.s.†
	$\%^{A\rightarrow G}$	30	-6.3 (-14.1, 2.0)		33	-5.9 (-13.0, 2.5)		n.s.	30	-7.3 (-25.1, 10.7)		29	-4.8 (-14.7, 4.4)		n.s.	n.s.
Holo-Transcobalamine [pmol/L]	0		88.6 (± 24.6)<br>85.6 (71.2, 102.0)	a		85.0 (± 35.3)	a†	n.s.†		89.0 (± 27.3)<br>90.2 (70.3, 99.7)	a		91.8 (79.0, 123.0)	a	n.s.	n.s.†
	10	29	85.1 (± 26.1)<br>87.6 (63.5, 97.0)	a	33	86.3 (± 31.9)	a†	n.s.†	30	80.9 (± 22.4)<br>78.7 (64.7, 100.2)	a	29	92.0 (± 31.9)<br>88.5 (65.4, 117.0)	a	n.s.†	n.s.†
	20		80.7 (63.3, 87.4)	a		79.1 (± 25.8)<br>79.5 (57.1, 97.4)	a†	n.s.		76.2 (61.6, 106.6)	a		91.5 (65.5, 111.0)	a	n.s.	n.s.
	$\%^{A\rightarrow F}$	29	-2.4 (± 21.3)		33	4.8 (± 19.3)		n.s.†	30	-6.1 (± 20.4)		31	-4.5 (± 18.9)		n.s.†	n.s.†
	$\%^{A\rightarrow G}$	30	-7.2 (-22.1, 5.7)		33	-8.0 (-19.6, 10.9)		n.s.	30	-5.0 (-18.3, 19.7)		29	-9.0 (-21.4, 9.6)		n.s.	n.s.
Vitamin B_1 [nmol/L]	0		144.5 (134.2, 150.7)	a		144.9 (± 25.5)<br>142.6 (125.9, 160.3)	a†	n.s.		135.3 (120.5, 148.4)	a,b		139.3 (126.3, 156.3)	a	n.s.	n.s.
	10	29	128.6 (117.4, 138.4)	b	33	131.6 (± 21.4)<br>129.8 (120.3, 145.3)	b†	n.s.	30	125.8 (117.5, 143.3)	a	29	134.8 (115.5, 151.1)	a	n.s.	n.s.
	20		150.0 (133.1, 155.6)	a		142.4 (± 26.1)<br>145.2 (118.2, 160.9)	a†	n.s.		145.1 (128.8, 159.8)	b		139.9 (124.5, 157.9)	a	n.s.	n.s.
	$\%^{A\rightarrow F}$	29	-10.7 (± 9.8)<br>-11.9 (-15.3, -4.7)		33	-8.5 (± 10.0)		n.s.†	30	-8.4 (-10.6, 4.1)		31	-6.3 (-12.9, 4.1)		n.s.	n.s.
	$\%^{A\rightarrow G}$	30	4.1 (± 17.3)		33	-1.0 (± 14.1)		n.s.†	30	8.2 (± 18.7)		29	2.3 (± 17.7)		n.s.†	n.s.†

Table S3. Continued.

Parameters	Week	*n*	HTGI Characteristics*	Δ	*n*	HTGC Characteristics*	Δ	◊	*n*	PDI Characteristics*	Δ	*n*	PDC Characteristics*	Δ	◊	●
Vitamin B_6 [nmol/L]	0		54.7 (44.5, 85.4)	a		48.3 (32.7, 82.5)	a	n.s.		47.9 (36.1, 65.6)	a		54.4 (42.9, 75.2)	a	n.s.	n.s.
	10	29	76.1 (56.4, 94.1)	b	33	52.5 (35.6, 83.1)	a	n.s.	30	58.1 (46.1, 80.9)	b	29	63.4 (51.6, 91.9)	b	n.s.	0.045
	20		58.8 (46.2, 74.9)	a		43.8 (35.4, 82.6)	a	n.s.		49.8 (36.6, 58.3)	a		47.9 (39.5, 59.0)	a	n.s.	n.s.
	$\%^{A\rightarrow F}$	29	18.8 (-12.4, 80.7)		33	18.0 (-14.5, 49.2)		n.s.	30	31.1 (-3.0, 60.8)		31	13.3 (4.2, 39.1)		n.s.	n.s.
	$\%^{A\rightarrow G}$	30	11.0 (± 45.9)<br>4.7 (-14.9, 45.6)		33	10.0 (± 42.4)		n.s.†	30	-3.4 (-19.4, 28.1)		29	-2.8 (-35.5, 19.8)		n.s.	n.s.
Vitamin A [µmol/L]	0		2.1 (± 0.3)<br>2.1 (1.9, 2.3)	a		2.0 (1.8, 2.5)	a	n.s.		2.0 (± 0.5)	a†		1.8 (± 0.4)	a†	n.s.†	n.s.†
	10	29	2.0 (± 0.4)<br>2.0 (1.8, 2.2)	a	33	2.1 (± 0.4)<br>2.1 (1.8, 2.3)	a	n.s.†	30	1.9 (± 0.4)	b†	29	1.9 (± 0.4)	b†	n.s.†	n.s.†
	20		2.0 (1.8, 2.2)	a		2.1 (1.8, 2.4)	a	n.s.		2.0 (± 0.4)<br>2.0 (1.7, 2.2)	a,b†		1.9 (± 0.4)	a,b†	n.s.†	n.s.
	$\%^{A\rightarrow F}$	29	-4.9 (± 13.0)<br>-4.3 (-14.5, 4.5)		33	0.9 (-5.9, 9.3)		n.s.	30	-5.4 (± 14.7)<br>-7.8 (-15.5, 2.5)		31	3.9 (-2.1, 12.4)		0.001	n.s.†
	$\%^{A\rightarrow G}$	30	-1.4 (± 14.8)		33	0.9 (± 16.6)		n.s.†	30	0.3 (± 16.5)		29	4.4 (± 15.9)		n.s.†	n.s.†
Vitamin D [nmol/L]	0		70.6 (± 24.9)<br>68.3 (52.8, 86.6)	a†		62.3 (43.6, 73.2)	a	0.032		56.4 (± 16.9)<br>55.7 (47.1, 71.4)	a†		58.5 (42.7, 75.1)	a	n.s.	0.013†
	10	29	76.4 (± 21.6)	a†	33	68.9 (± 14.2)<br>70.5 (62.9, 77.6)	b	n.s.†	30	63.6 (± 16.5)<br>59.2 (53.4, 75.7)	b†	29	67.0 (50.3, 89.9)	b	n.s.	0.013†
	20		63.7 (± 21.4)	b†		57.6 (± 13.6)<br>58.9 (48.4, 66.8)	a	n.s.†		56.6 (± 17.5)<br>56.3 (44.9, 64.1)	a†		53.2 (40.4, 67.9)	a	n.s.	n.s.†
	$\%^{A\rightarrow F}$	29	13.1 (± 22.9)<br>9.8 (-1.4, 26.6)		33	19.3 (± 24.8)		n.s.†	30	15.1 (-1.8, 29.4)		31	15.4 (3.2, 33.9)		n.s.	n.s.
	$\%^{A\rightarrow G}$	30	-14.9 (-19.9, 3.8)		33	-3.2 (-13.7, 9.2)		n.s.	30	1.8 (± 19.2)<br>-1.2 (-11.3, 15.8)		29	-2.6 (± 16.1)		n.s.†	0.027

Table S3. Continued.

Parameters	Week	*n*	HTGI Characteristics*	Δ	*n*	HTGC Characteristics*	Δ	◊	*n*	PDI Characteristics*	Δ	*n*	PDC Characteristics*	Δ	◊	●
Vitamin E [μmol/L]	0		42.3 (35.1, 44.5)	a		42.3 (± 10.8)<br>41.0 (34.3, 47.1)	a†	n.s.		34.5 (32.8, 41.0)	a		35.3 (± 8.2)<br>34.3 (29.0, 39.2)	a†	n.s.	0.015
	10	29	34.2 (31.9, 38.9)	b	33	43.7 (± 9.9)<br>42.3 (37.3, 48.4)	a†	0.003	30	32.8 (28.0, 35.8)	a	29	37.6 (± 7.9)<br>35.7 (32.4, 43.4)	b†	0.025	n.s.
	20		44.5 (35.4, 51.8)	a		45.7 (± 11.6)<br>44.9 (37.6, 55.1)	a†	n.s.		36.3 (30.5, 42.2)	a		38.8 (± 7.7)<br>38.0 (32.6, 42.2)	b†	n.s.	0.009
	$\%^{A\rightarrow F}$	29	-12.3 (± 14.9)<br>-9.9 (-21.0, -1.6)		33	1.3 (-6.7, 16.6)		<0.001	30	-7.8 (± 20.5)		31	8.7 (± 15.5)		0.001†	n.s.†
	$\%^{A\rightarrow G}$	30	2.3 (-7.1, 13.8)		33	5.1 (-3.7, 16.5)		n.s.	30	0.1 (-13.1, 12.2)		29	4.3 (1.0, 19.7)		n.s.	n.s.
Ferritin [μg/L]	0		133.3 (72.6, 221.2)	a		113.5 (58.5, 185.3)	a	n.s.		147.1 (76.7, 236.5)	a		161.9 (81.9, 200.5)	a	n.s.	n.s.
	10	29	111.6 (63.6, 164.2)	b	27	90.8 (47.2, 150.5)	b	n.s.	30	109.1 (59.6, 191.1)	b	28	138.4 (75.5, 201.9)	b	n.s.	n.s.
	20		119.2 (84.0, 186.6)	b		94.1 (52.4, 144.5)	b	n.s.		99.5 (63.6, 149.6)	b		138.4 (56.1, 186.4)	b	n.s.	n.s.
	$\%^{A\rightarrow F}$	29	-12.8 (± 24.8)<br>-17.2 (-30.9, 0.2)		33	-16.0 (-24.4, -3.2)		n.s.	30	-15.4 (± 25.3)		31	-10.6 (± 25.9)		n.s.†	n.s.†
	$\%^{A\rightarrow G}$	30	-11.5 (-20.8, -0.5)		33	-17.7 (-29.0, -12.6)		0.038	30	-26.0 (-34.5, -7.6)		29	-16.3 (-27.0, -9.3)		n.s.	n.s.
Transferrin [g/L]	0		2.7 (± 0.2)<br>2.7 (2.6, 2.8)	a		2.6 (2.4, 2.9)	a	n.s.		2.6 (± 0.4)<br>2.5 (2.3, 2.9)	a,b		2.5 (2.4, 2.6)	a	n.s.	n.s.†
	10	29	2.6 (± 0.3)<br>2.6 (2.3, 2.7)	b	27	2.7 (± 0.4)<br>2.6 (2.4, 2.9)	a	n.s.†	30	2.5 (± 0.4)<br>2.5 (2.2, 2.7)	a	28	2.6 (± 0.3)<br>2.5 (2.4, 2.7)	a,b	n.s.†	n.s.†
	20		2.7 (2.5, 2.8)	a		2.7 (2.5, 2.8)	a	n.s.		2.7 (2.4, 2.8)	b		2.6 (2.5, 2.8)	b	n.s.	n.s.
	$\%^{A\rightarrow F}$	29	-5.0 (± 7.6)		33	-0.6 (± 7.2)		0.023†	30	-2.8 (± 9.1)		31	0.3 (± 6.7)		n.s.†	n.s.†
	$\%^{A\rightarrow G}$	30	-0.6 (± 9.5)<br>0.0 (-7.4, 3.7)		33	0.0 (-3.6, 8.0)		n.s.	30	2.7 (± 8.6)		29	4.3 (± 8.4)		n.s.†	n.s.†

Table S3. Continued.

Parameters	Week	HTGI *n*	HTGI Characteristics*	HTGI Δ	HTGC *n*	HTGC Characteristics*	HTGC Δ	◊	PDI *n*	PDI Characteristics*	PDI Δ	PDC *n*	PDC Characteristics*	PDC Δ	◊	●
Transferrin saturation [%]	0		24.6 (20.1, 28.7)	a		22.4 (19.5, 27.6)	a	n.s.		24.6 (19.9, 29.1)	a		30.3 (26.0, 33.3)	a	0.020	n.s.
	10	29	21.1 (19.4, 26.4)	a	27	27.1 (21.7, 34.6)	a	n.s.	29	23.9 (19.1, 25.6)	a	28	26.4 (22.4, 33.6)	a	0.043	n.s.
	20		24.5 (19.5, 34.0)	a		24.0 (20.0, 29.1)	a	n.s.		26.0 (22.2, 29.4)	a		26.2 (22.5, 30.2)	a	n.s.	n.s.
	$\%^{A \to F}$	29	-2.8 (± 33.6)<br>-4.3 (-26.2, 18.2)		33	-0.0 (-15.5, 42.3)		n.s.	29	1.7 (± 29.3)		31	0.5 (± 28.8)		n.s.†	n.s.†
	$\%^{A \to G}$	30	8.2 (± 35.7)<br>8.3 (-18.2, 22.2)		33	0.2 (± 30.9)		n.s.†	29	4.3 (-8.3, 16.4)		29	-10.7 (-23.2, 2.8)		n.s.	n.s.

* Variables expressed as mean (± SD) and/or as median (25th, 75th percentile) depending on the statistical test that was performed; Δ Differences within groups comparing points in time, points in time without a common letter are significantly different, $p < 0.05$; ◊ Differences between each intervention group and their corresponding control group; ● Differences between both intervention groups; $\%^{A \to F}$, percentage change from baseline to week 10; $\%^{A \to G}$, percentage change from baseline to follow-up; † Calculated with parametric test. Abbreviations: HTGC, hypertriglyceridemia control; HTGI, hypertriglyceridemia intervention; PDC, prediabetes control; PDI, prediabetes intervention.

Table S4. Nutrient status in 24 h urine at baseline, after the intervention period and at follow-up.

Parameters	Week	*n*	HTGI Characteristics*	Δ	*n*	HTGC Characteristics*	Δ	◊	*n*	PDI Characteristics*	Δ	*n*	PDC Characteristics*	Δ	◊	●
Calcium 24h urine [mmol/24h]	0		4.3 (3.1, 6.5)	a		3.9 (± 1.6)<br>3.9 (2.6, 5.0)	a†	n.s.		4.1 (3.1, 5.0)	a		4.4 (± 1.8)<br>4.4 (2.9, 5.5)	a†	n.s.	n.s.
	10	29	4.2 (± 1.9)<br>3.9 (2.8, 5.4)	a	31	4.2 (± 1.9)	a†	n.s.†	29	4.5 (± 2.8)<br>4.2 (2.4, 5.8)	a	29	4.3 (± 2.2)	a†	n.s.†	n.s.†
	20		4.6 (± 1.8)<br>4.8 (3.2, 5.6)	a		4.2 (± 1.8)	a†	n.s.†		4.6 (2.3, 5.9)	a		4.1 (± 2.1)<br>4.3 (2.9, 5.0)	a†	n.s.	n.s.
	$\%^{A\rightarrow F}$	29	-8.5 (± 37.7)<br>-5.6 (-43.7, 18.5)		32	10.5 (± 38.6)		n.s.†	29	0.0 (-29.4, 16.7)		31	-7.1 (-25.8, 20.1)		n.s.	n.s.
	$\%^{A\rightarrow G}$	30	1.1 (± 38.3)<br>8.3 (-26.5, 30.1)		33	3.8 (-16.7, 28.3)		n.s.	30	10.1 (± 54.3)		29	-0.4 (± 46.5)		n.s.†	n.s.†
Chloride 24h urine [mmol/24h]	0		140.0 (97.0, 227.0)	a,b		139.0 (96.0, 179.0)	a	n.s.		122.0 (104.0, 164.0)	a		127.0 (98.5, 145.5)	a	n.s.	n.s.
	10	29	116.0 (81.0, 150.0)	a	31	129.0 (98.0, 163.0)	a	n.s.	29	144.6 (± 72.1)<br>138.0 (90.0, 170.0)	a	27	122.8 (± 44.8)<br>126.0 (88.0, 155.0)	a	n.s.†	n.s.
	20		130.0 (109.0, 204.0)	b		150.0 (108.0, 181.0)	a	n.s.		154.0 (113.0, 177.0)	a		123.0 (104.0, 175.5)	a	n.s.	n.s.
	$\%^{A\rightarrow F}$	29	-31.7 (-45.0, 18.6)		33	-9.0 (-26.3, 33.1)		n.s.	30	17.4 (± 55.4)<br>7.9 (-19.4, 55.2)		29	-1.1 (± 34.1)		n.s.†	0.040
	$\%^{A\rightarrow G}$	30	4.9 (-12.4, 40.9)		33	5.0 (-14.5, 34.5)		n.s.	29	6.8 (-14.7, 47.1)		28	10.9 (-5.3, 42.9)		n.s.	n.s.
Potassium 24h urine [mmol/24h]	0		70.0 (53.0, 80.0)	a		68.7 (± 22.2)<br>73.0 (54.0, 84.0)	a†	n.s.		67.4 (± 22.1)<br>71.0 (53.5, 81.0)	a†		71.6 (± 17.9)<br>74.0 (58.0, 80.0)	a	n.s.†	n.s.
	10	29	64.0 (57.0, 83.0)	a	31	69.8 (± 24.7)<br>70.0 (51.0, 81.0)	a†	n.s.	30	72.4 (± 26.1)<br>70.5 (55.0, 86.8)	a†	29	77.7 (± 24.1)<br>81.0 (66.0, 89.0)	a	n.s.†	n.s.
	20		69.0 (53.0, 78.0)	a		68.5 (± 22.5)<br>67.0 (56.0, 82.0)	a†	n.s.		76.9 (± 28.2)<br>73.0 (61.8, 91.3)	a†		77.0 (57.0, 91.0)	a	n.s.	n.s.
	$\%^{A\rightarrow F}$	29	12.4 (± 45.2)<br>0.0 (-15.3, 35.1)		33	7.8 (-21.6, 29.5)		n.s.	30	14.7 (± 39.4)<br>24.5 (-14.0, 41.7)		31	0.0 (-15.1, 28.4)		n.s.	n.s.†
	$\%^{A\rightarrow G}$	30	-4.6 (-29.9, 51.5)		33	11.5 (-21.1, 26.7)		n.s.	30	14.0 (-8.4, 54.7)		29	-1.3 (-12.4, 30.8)		n.s.	n.s.

Table S4. Continued.

Parameters	Week	*n*	HTGI<br>Characteristics*	Δ	*n*	HTGC<br>Characteristics*	Δ	◊	*n*	PDI<br>Characteristics*	Δ	*n*	PDC<br>Characteristics*	Δ	◊	●
Creatinine 24h urine [mmol/24h]	0		10.7 (8.6, 12.5)	a		10.1 (8.6, 12.0)	a	n.s.		10.1 (8.9, 12.7)	a		10.7 (8.4, 11.8)	a	n.s.	n.s.
	10	29	8.6 (7.5, 10.7)	b	31	10.9 (8.7, 13.2)	a	0.038	30	9.6 (8.2, 11.3)	a	29	11.3 (8.4, 13.5)	a	n.s.	n.s.
	20		9.0 (7.3, 11.5)	a,b		11.0 (8.7, 13.0)	a	n.s.		10.2 (8.9, 11.4)	a		10.4 (8.2, 13.9)	a	n.s.	n.s.
	$\%^{A\rightarrow F}$	29	-11.5 (± 23.6)		33	2.0 (± 28.4)		0.047†	30	-4.9 (± 16.5)<br>-7.7 (-12.3, 6.4)		31	1.1 (-12.8, 13.8)		n.s.	n.s.†
	$\%^{A\rightarrow G}$	30	-3.5 (-20.0, 4.1)		33	2.5 (-10.1, 15.3)		0.049	30	-2.3 (-13.0, 11.6)		29	-1.8 (-15.6, 18.6)		n.s.	n.s.
Magnesium 24h urine [mmol/24h]	0		3.9 (3.2, 5.1)	a		4.4 (± 1.6)<br>4.1 (3.2, 5.2)	a†	n.s.		4.1 (3.6, 5.0)	a		4.2 (± 1.1)<br>4.3 (3.2, 4.9)	a†	n.s.	n.s.
	10	29	3.3 (2.8, 3.9)	a	31	4.2 (± 1.3)<br>4.3 (3.5, 5.3)	a†	0.029	30	4.5 (3.9, 5.1)	a	28	4.6 (± 1.8)<br>4.4 (3.6, 5.3)	a†	n.s.	0.001
	20		3.9 (3.5, 4.9)	a		4.6 (± 1.7)<br>4.5 (3.6, 5.2)	a†	n.s.		4.5 (3.9, 5.9)	a		4.5 (± 1.8)<br>4.2 (3.4, 5.6)	a†	n.s.	n.s.
	$\%^{A\rightarrow F}$	29	-10.6 (± 31.2)<br>-8.3 (-34.1, 11.4)		32	-7.9 (-23.8, 17.9)		n.s.	30	12.2 (± 35.1)		31	5.7 (± 30.9)		n.s.†	0.011†
	$\%^{A\rightarrow G}$	29	6.3 (-23.4, 25.9)		32	2.5 (-19.4, 29.3)		n.s.	30	3.3 (-12.7, 39.5)		28	5.0 (-23.4, 29.9)		n.s.	n.s.
Sodium 24h urine [mmol/24h]	0		160.0 (113.0, 232.0)	a		150.0 (± 42.2)<br>146.0 (114.0, 184.0)	a†	n.s.		129.0 (101.0, 164.0)	a		130.5 (105.8, 158.0)	a	n.s.	n.s.
	10	29	136.0 (99.0, 155.0)	a	31	145.5 (± 53.0)<br>132.0 (109.0, 185.0)	a†	n.s.	29	152.6 (± 76.8)<br>135.0 (100.0, 196.0)	a	28	129.6 (± 55.7)<br>130.0 (89.5, 163.5)	a	n.s.†	n.s.
	20		158.0 (109.0, 221.0)	a		165.8 (± 71.7)<br>150.0 (113.0, 204.0)	a†	n.s.		175.0 (117.0, 199.0)	a		139.5 (118.5, 186.5)	b	n.s.	n.s.
	$\%^{A\rightarrow F}$	29	-28.6 (-45.6, 11.5)		33	-8.4 (-23.3, 22.4)		n.s.	30	17.6 (± 54.0)<br>12.8 (-19.1, 56.1)		30	-4.0 (± 35.1)		n.s.†	0.018
	$\%^{A\rightarrow G}$	30	0.4 (-14.5, 26.5)		33	4.7 (-18.6, 42.1)		n.s.	29	22.8 (-12.8, 43.4)		28	13.7 (-3.9, 50.7)		n.s.	n.s.

Table S4. Continued.

Parameters	Week	*n*	HTGI<br>Characteristics*	Δ	*n*	HTGC<br>Characteristics*	Δ	◊	*n*	PDI<br>Characteristics*	Δ	*n*	PDC<br>Characteristics*	Δ	◊	●
Selenium 24h urine [μmol/24h]	0		0.3 (0.2, 0.4)	a		0.3 (0.2, 0.3)	a	n.s.		0.2 (0.2, 0.3)	a		0.3 (0.2, 0.3)	a,b	0.035	0.004
	10	29	0.2 (0.2, 0.3)	b	31	0.3 (0.2, 0.3)	a	n.s.	29	0.2 (0.2, 0.3)	a	29	0.3 (0.3, 0.4)	a	n.s.	n.s.
	20		0.3 (0.2, 0.3)	b		0.3 (0.2, 0.4)	a	n.s.		0.26 (0.2, 0.3)	a		0.3 (0.2, 0.3)	b	n.s.	n.s.
	$\%^{A\rightarrow F}$	29	-15.4 (-30.3, 2.1)		33	7.1 (-14.8, 27.0)		0.042	29	5.9 (-8.7, 31.3)		31	3.4 (-16.6, 44.2)		n.s.	0.016
	$\%^{A\rightarrow G}$	30	-13.0 (-27.4, 9.7)		33	2.4 (-21.7, 46.2)		n.s.	30	22.9 (± 48.1)<br>17.4 (-12.6, 44.8)		29	-4.7 (± 31.0)		0.011†	0.015
Zinc 24h urine [μmol/24h]	0		7.6 (4.7, 12.6)	a		7.6 (5.2, 10.3)	a	n.s.		10.3 (± 4.5)<br>10.1 (7.3, 12.2)	a		7.6 (± 2.9)<br>6.9 (5.9, 10.2)	a	0.010†	n.s.
	10	29	7.4 (± 4.1)<br>7.0 (5.2, 9.0)	a	31	9.1 (± 4.6)<br>8.8 (4.8, 11.0)	a	n.s.†	29	10.8 (± 4.4)<br>10.6 (8.1, 13.9)	a	29	7.5 (5.6, 9.8)	a	0.041	0.004†
	20		5.6 (3.8, 12.0)	a		9.0 (7.1, 11.2)	a	n.s.		8.8 (7.1, 13.0)	a		8.4 (6.7, 10.5)	a	n.s.	n.s.
	$\%^{A\rightarrow F}$	29	-25.0 (-43.9, 22.4)		33	1.3 (-31.8, 44.7)		n.s.	29	-3.9 (-21.1, 25.5)		31	2.2 (-17.2, 62.5)		n.s.	n.s.
	$\%^{A\rightarrow G}$	30	-24.1 (-43.1, 34.3)		33	11.4 (-23.8, 58.6)		n.s.	30	-16.8 (-38.6, 27.8)		29	14.7 (-14.1, 53.8)		n.s.	n.s.

* Variables expressed as mean (± SD) and/or as median (25th, 75th percentile) depending on the statistical test that was performed; Δ Differences within groups comparing points in time, points in time without a common letter are significantly different, $p < 0.05$; ◊ Differences between each intervention group and their corresponding control group; ● Differences between both intervention groups; $\%^{A\rightarrow F}$, percentage change from baseline to week 10; $\%^{A\rightarrow G}$, percentage change from baseline to follow-up; † Calculated with parametric test. Abbreviations: HTGC, hypertriglyceridemia control; HTGI, hypertriglyceridemia intervention; PDC, prediabetes control; PDI, prediabetes intervention.
